# Tobacco-Attributable Age-Related Macular Degeneration Vision Impairment in Japan: A National and Prefecture-Level Analysis From 1990 to 2040

**DOI:** 10.1167/tvst.15.2.23

**Published:** 2026-02-18

**Authors:** Luoming Huang, Li Cong, Andrzej Grzybowski

**Affiliations:** 1 Department of Ophthalmology and Optometry, The School of Medical Technology and Engineering, Fujian Medical University, Fuzhou, Fujian Province, China; 2 Faculty of Comprehensive Rehabilitation, Hiroshima International University, Hiroshima, Japan; 3 Institute for Research in Ophthalmology, Foundation for Ophthalmology Development, Poznan, Poland

**Keywords:** age-related macular degeneration, Japan, global burden of disease, tobacco, vision impairment

## Abstract

**Purpose:**

This study aimed to analyze the spatiotemporal burden of vision impairment due to age-related macular degeneration (AMD) in Japan from 1990 to 2021, projecting to 2040.

**Methods:**

Using data from the Global Burden of Disease (GBD) study, we systematically analyzed the prevalence, disability-adjusted life years (DALYs), and temporal trends of vision impairment due to AMD in Japan from 1990 to 2021 and projected disease burden to 2040. We also quantified the burden of vision impairment due to AMD attributable to tobacco.

**Results:**

From 1990 to 2021, the number of AMD-related vision impairment cases increased by 159% to 93,310 (95% uncertainty interval [UI], 78,103–112,033); DALYs increased by 134% to 8907 (95% UI, 5984–12,298). However, the age-standardized prevalence rates declined from 22.3 to 19.73 per 100,000, and DALY rates decreased from 2.34 to 1.94. Prevalence was slightly higher in females, although the DALY rate difference was minimal. Burden disparities across all 47 prefectures were small. The contribution of tobacco to age-standardized DALYs decreased by 36.3%. Projections to 2040 estimate a 42.51% increase in total cases, with a rise in the age-standardized prevalence rate among males but a decline continuing among females.

**Conclusions:**

Despite an increase in absolute cases, age-standardized rates of AMD-related vision impairment in Japan declined from 1990 to 2021, likely due to universal health insurance coverage and effective tobacco control policies. This supports ongoing investment to alleviate the burden in an aging society.

**Translational Relevance:**

Translating 30-year population data, this study shows that tobacco control and healthcare equity reduce AMD-related vision impairment burden, informing vision loss prevention in aging societies.

## Introduction

Age-related macular degeneration (AMD) is one of the leading causes of irreversible vision loss and blindness among older adults worldwide.^1^ With the accelerating aging of the global population, its prevalence continues to rise, imposing a substantial economic burden on healthcare systems.^2^ Approximately 30% of the elderly population globally is affected by AMD, which accounts for about 8.7% of all blindness cases and causes an estimated 500,000 individuals to lose their sight each year.^3^^,^^4^ Findings from the Global Burden of Disease (GBD) study indicate that AMD has become a major cause of visual impairment in regions with a middle to high socio-demographic index (SDI).^5^ Therefore, accurately assessing the potential impact of AMD on individual health and societal outcomes is particularly crucial in developed countries, especially within aging societies.

AMD is a multifactorial disease whose pathogenesis involves complex interactions among aging, environmental risk factors, and genetic susceptibility.^1^ Among these, aging is the most significant risk factor. Epidemiological data indicate that the incidence of advanced AMD doubles approximately every decade after the age of 60,^6^ suggesting a continuing increase in the future disease burden. Among modifiable risk factors, smoking has been identified by multiple prospective cohort studies as an independent and significant risk factor, with a clear dose–response relationship.^7^^–^^10^ Smoking exacerbates the process of retinal damage by enhancing oxidative stress and inflammatory responses, thereby promoting the onset and progression of macular degenerative changes.^11^

As one of the most rapidly aging countries globally, Japan's trends in AMD prevalence and associated vision impairment hold significant public health implications and serve as a representative case for investigation. However, although several studies have reported epidemiological characteristics of AMD in various countries, comprehensive, long-term, and multiregional analyses of its disease burden in Japan remain limited. Utilizing data from the GBD, this study systematically evaluated the prevalence and disability-adjusted life years (DALYs) of AMD-related vision impairment in Japan from 1990 to 2021 and quantified the tobacco-related burden of vision impairment attributable to AMD. The aim was to provide a more comprehensive and in-depth understanding of the burden characteristics of AMD-related vision impairment across Japan.

## Methods

### Data Sources

Data were obtained from the GBD 2021 database maintained by the Institute for Health Metrics and Evaluation (IHME). GBD 2021 employs standardized methods to estimate incidence, prevalence, mortality, and DALYs for 371 diseases and injuries across 204 countries/territories, encompassing 288 causes of death and 88 risk factors, with subnational modeling for 21 regions. Data were synthesized from multiple heterogeneous sources, including peer-reviewed literature, census records, disease registries, clinical electronic health records, and epidemiological surveillance reports, standardized using Bayesian meta-regression tools (DisMod-MR 2.1).^12^ Dataset access complied with the IHME non-commercial use agreement. The GBD study utilizes a publicly available secondary database containing no personally identifiable information. This research adhered to U.S. Public Law 112-74 and 45 CFR 46 and thus was exempt from institutional review board approval.

The definition of AMD pertains to age-related deterioration of the macula, the central portion of the retina responsible for central vision, resulting in central vision loss.^13^ Vision impairment was categorized according to the *International Classification of Diseases*, 11th Revision, which defines three severity levels: moderate vision loss (visual acuity ≥ 6/60 and < 6/18), severe vision loss (visual acuity ≥ 3/60 and < 6/60), and blindness (visual acuity < 3/60 or a visual field of less than 10° around central fixation). The GBD estimates for AMD quantify the burden specifically associated with the vision-impairing sequelae of the disease (moderate vision loss, severe vision loss, and blindness), not the prevalence of asymptomatic or early-stage AMD.^13^ Following the comparative risk assessment (CRA) framework,^14^ the burden of vision impairment due to AMD attributable to tobacco was quantified.

### Risk Factor

The estimation of tobacco-attributable burden was conducted within the refined CRA framework of the GBD 2021 study. Compared to prior iterations, GBD 2021 introduced key methodological advancements for estimating risk exposure, relative risks (RRs), and attributable burdens. For 211 risk–outcome pairs, burden-of-proof risk function (BPRF) analyses were employed to address unexplained heterogeneity across input studies, yielding a more conservative and evidence-graded interpretation of associations. The strength of evidence for each pair is summarized by a star-rating system. For AMD, tobacco use—categorized as a level-one behavioral risk—was the sole risk factor that met the stringent GBD inclusion criteria for quantification, remaining a validated risk factor through successive GBD cycles. Within this framework, exposure to tobacco encompasses current and former use of smoked tobacco products.^13^ The theoretical minimum risk exposure level (TMREL) is defined as never smoking. Population-level exposure distributions from 1990 to 2021 were estimated by synthesizing survey and sales data using spatiotemporal Gaussian process regression. The dose–response RRs for the association between smoked tobacco use and AMD-related vision impairment were sourced from the GBD 2021 systematic reviews, which applied the aforementioned BPRF methodology.^13^

### Autoregressive Integrated Moving Average Forecasting Model

We employed autoregressive integrated moving average (ARIMA[p,d,q]) models to analyze long-term trends and project the future burden of AMD-attributable vision impairment through 2040. The optimal model orders (p, d, q) for each sex-specific series were automatically selected using the auto.arimafunction in R (R Foundation for Statistical Computing, Vienna, Austria), which minimizes the Akaike information criterion and ensures stationarity. For age-standardized prevalence rates, the selected model was ARIMA(0,2,1). This model structure implies that the second differences of the time series are stationary and follow a process driven by a first-order moving average component. The model can be expressed as (1 – *B*)^2^*Y*_*t* = (1 + θ*B*)ε_*t*, where *B* is the backshift operator; *Y*_*t* is the observed rate at time *t*; θ is the moving average coefficient, MA(1); and ε_*t* is white noise error. The sign and significance of MA(1) are critical for interpreting forecast directions, as a significant negative MA(1) term can indicate momentum reversion. Model adequacy was rigorously verified. Residual independence was confirmed via Ljung–Box tests, and normality of residuals was assessed, ensuring the robustness and validity of the long-term projections.

### Statistical Analysis

All of the age-standardized rates were extracted from GBD datasets. Analyses were comprised of three primary components: (1) analysis of prevalence and DALYs, including case counts and age-standardized rates for vision impairment due to AMD and its smoking-attributable component, with percentage changes between 1990 to 2021; (2) stratified trend analysis of age-standardized DALY rates for AMD-related vision impairment and its smoking-attributable component (1990–2021), stratified by 5-year age groups (45–49 to ≥95 years), gender, and prefecture; and (3) application of ARIMA time-series models to analyze historical trends and project the future burden (prevalence cases and rates) of AMD-related vision impairment from 2022 to 2040. All analyses were performed using RStudio 4.3.3, with statistical significance defined as two-sided *P* < 0.05. Key metrics are reported with their corresponding 95% uncertainty intervals (UIs).

## Results

### Burden of Vision Impairment Due to AMD

From 1990 to 2021, the number of vision impairment cases due to AMD showed an upward trend, whereas both the age-standardized prevalence rate and the age-standardized DALY rate exhibited a decline. The national number of prevalent cases increased from 36,027 (95% UI, 29,884–43,516) in 1990 to 93,310 (95% UI, 78,103–112,033) in 2021. However, the age-standardized prevalence rate decreased from 22.3 (95% UI, 18.62–26.85) per 100,000 population to 19.73 (95% UI, 16.56–23.9) per 100,000 population, with a percentage change (PC) of −11.52% (95% UI, −14.1 to −9.13) (Table; Supplementary Tables S1, S2). Over the same period, the total number of DALYs increased from 3814 (95% UI, 2442–5349) to 8907 (95% UI, 5984–12,298); yet, the age-standardized DALY rate declined from 2.34 (95% UI, 1.51–3.28) to 1.94 (95% UI, 1.27–2.66) per 100,000 population, with a PC of −17.25% (95% UI, −21.25 to −13.38) (Table; Supplementary Table S3).

**Table.** Age-Standardized Rates Per 100,000 of the Population and Percentage Change of Prevalence and DALYs of Vision Impairment Due to AMD From 1990 to 2021

	Prevalence Per 100,000 Population (95% UI)			DALYs (95% UI)		
	1990	2021	Change Between 1990 and 2021 (%)	1990	2021	Change Between 1990 and 2021 (%)
Global	99.5 (83.16 to 118.04)	94 (78.32 to 114.42)	−5.53 (−8.84 to −2.23)	8.38 (5.7 to 11.53)	6.78 (4.7 to 9.32)	−19.09 (−22.56 to −15.42)
Japan	22.3 (18.62 to 26.85)	19.73 (16.56 to 23.9)	−11.52 (−14.1 to −9.13)	2.34 (1.51 to 3.28)	1.94 (1.27 to 2.66)	−17.25 (−21.25 to −13.38)
Sex						
Male	21.84 (18.28 to 26.55)	19.44 (16.31 to 23.59)	−10.97 (−13.39 to −8.34)	2.34 (1.51 to 3.29)	1.97 (1.29 to 2.72)	−16.03 (−20.75 to −11.26)
Female	22.56 (18.85 to 26.97)	19.89 (16.58 to 23.83)	−11.82 (−14.65 to −9.06)	2.33 (1.51 to 3.3)	1.91 (1.26 to 2.63)	−18.4 (−23.13 to −13.24)
Age (y)						
45–49	1.68 (0.81 to 3.08)	1.39 (0.69 to 2.5)	−17.35 (−28.02 to −4.12)	0.23 (0.09 to 0.49)	0.17 (0.07 to 0.37)	−24.76 (−35.42 to −12.53)
50–54	10.85 (6.39 to 16.6)	8.88 (5.23 to 13.52)	−18.18 (−25.03 to −9.35)	1.49 (0.71 to 2.58)	1.11 (0.53 to 1.94)	−25.54 (−32.03 to −18.02)
55–59	27.86 (18.86 to 39.29)	23.17 (15.91 to 31.85)	−16.83 (−21.88 to −10.83)	3.51 (1.9 to 5.86)	2.71 (1.44 to 4.39)	−22.81 (−37.28 to −4.17)
60–64	52.18 (35.83 to 71.61)	44.6 (31.38 to 60.61)	−14.53 (−19 to −9.28)	6.25 (3.45 to 10.04)	4.95 (2.68 to 7.88)	−20.76 (−31.41 to −8.58)
65–69	86.81 (65.07 to 116.11)	75.78 (57.2 to 100.15)	−12.7 (−16.56 to −8.55)	9.66 (5.68 to 14.54)	7.81 (4.8 to 11.59)	−19.14 (−29.09 to −9.01)
70–74	137.74 (105.86 to 177.86)	121.6 (93.45 to 156.24)	−11.72 (−15.32 to −8.27)	14.38 (8.96 to 21.72)	11.81 (7.27 to 17.37)	−17.85 (−26.2 to −7.72)
75–79	221.36 (171.27 to 285.49)	196.78 (151.5 to 252.82)	−11.11 (−14.65 to −8.33)	22.3 (14.48 to 32.64)	18.57 (12.04 to 26.88)	−16.73 (−25.02 to −7.12)
80–84	336.49 (260.25 to 432.19)	301.1 (233.04 to 383.28)	−10.52 (−13.55 to −7.86)	33.31 (21.26 to 49.75)	28 (18.08 to 41.22)	−15.94 (−21.83 to −9.36)
85–89	497.98 (390.78 to 636.48)	450.08 (350.11 to 570.36)	−9.62 (−12.18 to −6.96)	47.94 (30.22 to 69.76)	41.15 (26.76 to 59.5)	−14.16 (−18.92 to −9.08)
90–94	713.93 (558.61 to 924.75)	653.71 (505.23 to 850.16)	−8.44 (−10.63 to −6)	68.1 (43.45 to 99.81)	60.01 (39.48 to 87.48)	−11.88 (−16.51 to −6.97)
95+	1051.47 (777.18 to 1394.17)	978.28 (731.05 to 1290.56)	−6.96 (−9.06 to −4.7)	101.68 (63.29 to 157.56)	92.09 (56.38 to 142.82)	−9.43 (−13.1 to −5.19)
Prefecture						
Hokkaido	22.48 (18.7 to 27.42)	19.84 (16.5 to 24.12)	−11.77 (−16.18 to −6.67)	2.38 (1.55 to 3.33)	1.96 (1.3 to 2.77)	−17.73 (−29.96 to −3.74)
Aomori	22.79 (19 to 27.67)	19.97 (16.61 to 23.99)	−12.35 (−17.15 to −7.58)	2.42 (1.58 to 3.4)	1.96 (1.27 to 2.79)	−18.78 (−31.11 to −3.45)
Iwate	22.68 (18.84 to 27.47)	19.99 (16.72 to 23.99)	−11.85 (−16.68 to −7.22)	2.4 (1.56 to 3.39)	1.96 (1.29 to 2.7)	−18.12 (−30.53 to −2.73)
Miyagi	22.31 (18.8 to 27.1)	19.71 (16.44 to 23.78)	−11.63 (−16.31 to −7.16)	2.34 (1.47 to 3.35)	1.92 (1.26 to 2.69)	−17.83 (−30.17 to −3.33)
Akita	22.58 (18.77 to 27.2)	20.02 (16.75 to 24.05)	−11.31 (−16.12 to −6.49)	2.38 (1.57 to 3.37)	1.98 (1.29 to 2.79)	−16.79 (−29.76 to −3.3)
Yamagata	22.26 (18.64 to 27.01)	19.9 (16.49 to 23.95)	−10.6 (−15.06 to −5.4)	2.33 (1.52 to 3.37)	1.94 (1.25 to 2.78)	−16.69 (−30.74 to −1.81)
Fukushima	22.54 (18.68 to 27.4)	19.96 (16.65 to 24.1)	−11.45 (−16.68 to −6.34)	2.38 (1.54 to 3.41)	1.96 (1.29 to 2.78)	−17.86 (−29.89 to −4.38)
Ibaraki	22.54 (18.79 to 27.01)	19.85 (16.58 to 24.05)	−11.91 (−16.53 to −7.23)	2.38 (1.52 to 3.36)	1.95 (1.26 to 2.73)	−17.8 (−30.56 to −3.47)
Tochigi	22.54 (18.9 to 26.81)	19.86 (16.53 to 23.94)	−11.86 (−15.91 to −7.23)	2.37 (1.5 to 3.34)	1.96 (1.26 to 2.79)	−17.29 (−28.87 to −3.88)
Gunma	22.38 (18.66 to 27)	19.85 (16.52 to 23.82)	−11.3 (−15.94 to −5.96)	2.34 (1.47 to 3.27)	1.94 (1.27 to 2.67)	−16.96 (−29.65 to −3.15)
Saitama	22.42 (18.68 to 27.2)	19.75 (16.49 to 23.9)	−11.89 (−16.1 to −6.79)	2.35 (1.5 to 3.33)	1.93 (1.27 to 2.75)	−17.71 (−29.89 to −3.01)
Chiba	22.25 (18.53 to 27.17)	19.8 (16.35 to 23.95)	−11.03 (−15.25 to −6.19)	2.33 (1.51 to 3.33)	1.94 (1.29 to 2.71)	−16.97 (−28.91 to −2.05)
Tokyo	22.05 (18.35 to 26.45)	19.6 (16.11 to 23.55)	−11.11 (−16.23 to −5.85)	2.29 (1.48 to 3.22)	1.91 (1.26 to 2.7)	−16.71 (−29.19 to −3.64)
Kanagawa	22.12 (18.39 to 26.65)	19.61 (16.25 to 23.69)	−11.31 (−15.79 to −6.15)	2.3 (1.47 to 3.32)	1.92 (1.25 to 2.65)	−16.3 (−28.26 to −2.58)
Niigata	22.16 (18.62 to 26.83)	19.8 (16.58 to 23.97)	−10.67 (−15.41 to −6.31)	2.31 (1.51 to 3.27)	1.94 (1.27 to 2.8)	−15.94 (−28.36 to −2.15)
Toyama	22.3 (18.46 to 27.21)	19.75 (16.51 to 23.72)	−11.4 (−15.97 to −5.94)	2.34 (1.52 to 3.37)	1.94 (1.27 to 2.76)	−17.35 (−28.82 to −4.58)
Ishikawa	22.31 (18.61 to 26.96)	19.8 (16.48 to 23.85)	−11.25 (−15.98 to −5.83)	2.34 (1.47 to 3.32)	1.94 (1.24 to 2.7)	−16.88 (−29.62 to −3.49)
Fukui	22.23 (18.43 to 26.65)	19.72 (16.42 to 23.6)	−11.31 (−15.91 to −6.29)	2.33 (1.48 to 3.34)	1.93 (1.24 to 2.74)	−17.18 (−30.41 to −2.27)
Yamanashi	22.38 (18.59 to 26.92)	19.79 (16.5 to 23.87)	−11.59 (−16.4 to −6.8)	2.35 (1.55 to 3.3)	1.94 (1.24 to 2.73)	−17.47 (−29.54 to −2.79)
Nagano	22.05 (18.3 to 26.44)	19.67 (16.33 to 23.76)	−10.79 (−15.93 to −5.55)	2.3 (1.47 to 3.25)	1.93 (1.25 to 2.68)	−16.11 (−28.66 to −1.4)
Gifu	21.26 (17.88 to 25.77)	19.74 (16.41 to 23.64)	−7.16 (−11.69 to −2.36)	2.17 (1.38 to 3.08)	1.93 (1.24 to 2.72)	−11.24 (−23.92 to 2.58)
Shizuoka	22.25 (18.59 to 26.96)	19.71 (16.34 to 23.68)	−11.4 (−16.23 to −6.22)	2.33 (1.5 to 3.38)	1.92 (1.22 to 2.72)	−17.55 (−30.1 to −2.29)
Aichi	21.13 (17.2 to 25.8)	18.62 (15.28 to 22.58)	−11.91 (−17.04 to −6.42)	2.31 (1.45 to 3.3)	1.89 (1.22 to 2.64)	−18.1 (−29.92 to −2.88)
Mie	22.33 (18.61 to 27.03)	19.79 (16.51 to 23.69)	−11.39 (−15.84 to −6.21)	2.34 (1.51 to 3.33)	1.93 (1.26 to 2.75)	−17.59 (−29.93 to −3.62)
Shiga	22.26 (18.71 to 27.09)	19.59 (16.34 to 23.6)	−12 (−16.91 to −7.28)	2.32 (1.48 to 3.35)	1.92 (1.26 to 2.65)	−17.25 (−29.33 to −3.09)
Kyoto	22.23 (18.49 to 26.9)	19.67 (16.42 to 23.8)	−11.52 (−16.17 to −6.59)	2.33 (1.48 to 3.31)	1.92 (1.24 to 2.7)	−17.77 (−30.1 to −3.95)
Osaka	22.56 (18.68 to 27.25)	19.92 (16.52 to 24.16)	−11.69 (−16.67 to −6.7)	2.38 (1.54 to 3.38)	1.97 (1.3 to 2.74)	−17.42 (−29.28 to −3.26)
Hyogo	22.45 (18.7 to 27.11)	19.75 (16.28 to 23.65)	−12.06 (−16.68 to −6.89)	2.36 (1.51 to 3.31)	1.93 (1.22 to 2.68)	−18.12 (−29.6 to −4)
Nara	22.29 (18.6 to 27)	19.79 (16.5 to 23.75)	−11.21 (−15.64 to −6.25)	2.34 (1.51 to 3.27)	1.94 (1.27 to 2.77)	−17.16 (−28.19 to −1.72)
Wakayama	22.48 (18.77 to 27.16)	19.94 (16.63 to 23.85)	−11.27 (−15.68 to −6.77)	2.36 (1.53 to 3.4)	1.96 (1.27 to 2.76)	−17.12 (−28.58 to −3.13)
Tottori	22.42 (18.78 to 26.99)	19.82 (16.64 to 24.1)	−11.59 (−16.06 to −6.85)	2.35 (1.47 to 3.34)	1.95 (1.25 to 2.76)	−17.03 (−29.3 to −0.42)
Shimane	22.43 (18.74 to 27.08)	19.9 (16.6 to 24.1)	−11.27 (−16.25 to −6.83)	2.36 (1.49 to 3.38)	1.95 (1.27 to 2.73)	−17.45 (−29.5 to −2.72)
Okayama	22.43 (18.72 to 27.31)	19.75 (16.39 to 23.78)	−11.94 (−16.46 to −7.32)	2.34 (1.5 to 3.32)	1.93 (1.26 to 2.65)	−17.47 (−28.97 to −2.69)
Hiroshima	22.25 (18.6 to 26.95)	19.76 (16.4 to 23.57)	−11.19 (−15.88 to −6.4)	2.32 (1.51 to 3.28)	1.93 (1.27 to 2.78)	−16.72 (−29.42 to −2.38)
Yamaguchi	22.47 (18.74 to 27.11)	19.94 (16.65 to 23.99)	−11.26 (−16.23 to −5.92)	2.37 (1.51 to 3.36)	1.97 (1.26 to 2.76)	−17.11 (−28.6 to −1.87)
Tokushima	22.54 (18.82 to 27.21)	19.92 (16.6 to 23.87)	−11.61 (−15.74 to −6.55)	2.37 (1.55 to 3.31)	1.97 (1.25 to 2.78)	−16.88 (−28.77 to −2.61)
Kagawa	22.37 (18.7 to 26.93)	19.83 (16.61 to 23.86)	−11.35 (−15.86 to −6.89)	2.34 (1.46 to 3.28)	1.95 (1.27 to 2.75)	−16.69 (−27.44 to −2.16)
Ehime	22.45 (18.71 to 27.05)	19.94 (16.75 to 24.14)	−11.18 (−16.03 to −6.5)	2.37 (1.52 to 3.34)	1.96 (1.26 to 2.75)	−17.16 (−29.09 to −3.83)
Kochi	22.69 (19.1 to 27.12)	20.02 (16.68 to 23.94)	−11.74 (−16.47 to −7.16)	2.4 (1.57 to 3.43)	1.98 (1.27 to 2.74)	−17.78 (−29.86 to −3.9)
Fukuoka	22.46 (18.6 to 27.08)	19.8 (16.43 to 23.99)	−11.84 (−16.65 to −6.83)	2.36 (1.49 to 3.38)	1.94 (1.27 to 2.75)	−17.82 (−31.56 to −3.6)
Saga	22.57 (18.73 to 27.27)	19.87 (16.45 to 23.91)	−11.95 (−17.23 to −7.29)	2.38 (1.56 to 3.34)	1.95 (1.26 to 2.75)	−18.01 (−31.13 to −4.73)
Nagasaki	22.64 (18.91 to 27.28)	19.92 (16.73 to 23.88)	−12.03 (−16.61 to −6.69)	2.4 (1.55 to 3.44)	1.96 (1.28 to 2.76)	−18.29 (−30.23 to −5.78)
Kumamoto	22.5 (18.77 to 27.23)	19.88 (16.63 to 23.68)	−11.65 (−16.58 to −6.1)	2.36 (1.53 to 3.32)	1.95 (1.28 to 2.72)	−17.31 (−29.83 to −3.18)
Oita	22.41 (18.52 to 27.12)	19.77 (16.57 to 23.58)	−11.75 (−16.55 to −7.1)	2.35 (1.52 to 3.37)	1.93 (1.27 to 2.66)	−17.92 (−30.33 to −2.16)
Miyazaki	22.64 (18.76 to 27.46)	19.92 (16.66 to 24)	−12.02 (−16.39 to −7.23)	2.37 (1.58 to 3.4)	1.95 (1.29 to 2.73)	−17.78 (−30.46 to −3.6)
Kagoshima	22.76 (18.85 to 27.55)	19.89 (16.65 to 24.1)	−12.62 (−17.05 to −7.52)	2.41 (1.55 to 3.44)	1.97 (1.29 to 2.76)	−18.42 (−30.42 to −5.41)
Okinawa	22.21 (18.38 to 26.8)	20.69 (17.29 to 24.98)	−6.85 (−11.17 to −2.06)	2.32 (1.48 to 3.33)	2.09 (1.34 to 2.96)	−10.18 (−23.75 to 4.32)

DALYs, disability-adjusted life years; UI, uncertainty interval.

**Figure 1.**
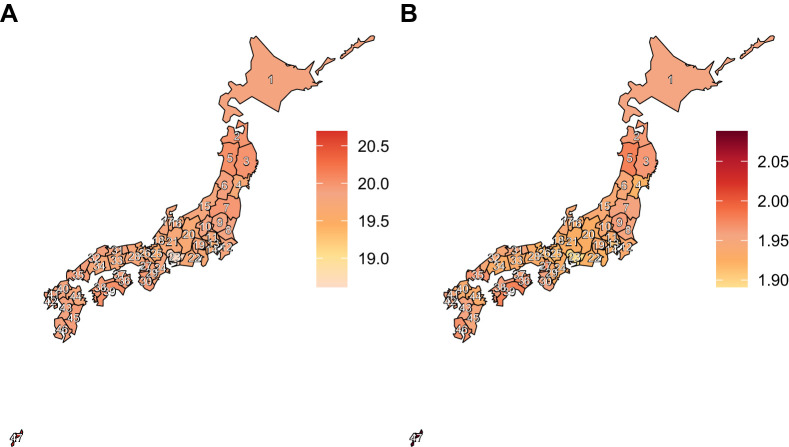
Distribution of vision impairment due to age-related macular degeneration in both sexes in Japan in 2021. (**A, B**) Age-standardized prevalence (**A**) and DALY rate (**B**) per 100,000 of the population in 2021. Prefectures are numbered from 1 to 47: 1, Hokkaido; 2, Aomori; 3, Iwate; 4, Miyagi; 5, Akita; 6, Yamagata; 7, Fukushima; 8, Ibaraki; 9, Tochigi; 10, Gunma; 11, Saitama; 12, Chiba; 13, Tokyo; 14, Kanagawa; 15, Niigata; 16, Toyama; 17, Ishikawa; 18, Fukui; 19, Yamanashi; 20, Nagano; 21, Gifu; 22, Shizuoka; 23, Aichi; 24, Mie; 25, Shiga; 26, Kyoto; 27, Osaka; 28, Hyogo; 29, Nara; 30, Wakayama; 31, Tottori; 32, Shimane; 33, Okayama; 34, Hiroshima; 35, Yamaguchi; 36, Tokushima; 37, Kagawa; 38, Ehime; 39, Kochi; 40, Fukuoka; 41, Saga; 42, Nagasaki; 43, Kumamoto; 44, Oita; 45, Miyazaki; 46, Kagoshima; 47, Okinawa.

Across all 47 prefectures of Japan, both the age-standardized prevalence rate and age-standardized DALY rate showed a consistent downward trend, with little regional variation (Fig. 1). In 2021, the prevalence rate ranged between 16.58 and 24.98 per 100,000 population, and the DALY rate ranged from 1.89 to 2.09 per 100,000. The greatest decline in the age-standardized prevalence rate was observed in Kagoshima Prefecture (−12.62%; 95% UI, −17.05 to −7.52), and the smallest in Okinawa Prefecture (−6.85%; 95% UI, −11.17 to −2.06). Similarly, the age-standardized DALY rate decreased most rapidly in Kagoshima Prefecture (−18.42%; 95% UI, −30.42 to −5.41) and least rapidly in Okinawa Prefecture (−10.18%; 95% UI, −30.42 to −5.41) (Table; Supplementary Table S1).

### Burden of Vision Impairment Due to AMD According to Sex and Age

From 1990 to 2021, the burden of vision impairment due to AMD showed consistent declines in both the age-standardized prevalence rate and age-standardized DALY rate among males and females, with all metrics slightly higher in females than in males. By sex, the age-standardized prevalence rate in males decreased from 21.84 (95% UI, 18.28–26.55) to 19.44 (95% UI, 16.31–23.59) per 100,000 population, with a PC of −10.97% (95% UI, −13.39 to −8.34). Among females, it declined from 22.56 (95% UI, 18.85–26.97) to 19.89 (95% UI, 16.58–23.83), with a PC of −11.82% (95% UI, −14.65 to −9.06). The age-standardized DALY rate decreased from 2.34 (95% UI, 1.51–3.29) to 1.97 (95% UI, 1.29–2.72) in males, with a PC of −16.03% (95% UI, −20.75 to −11.26), and from 2.33 (95% UI, 1.51–3.30) to 1.91 (95% UI, 1.26–2.63) in females, with a PC of −18.40% (95% UI, −23.13 to −13.24) (Table; Supplementary Tables S4, S5).

**Figure 2.**
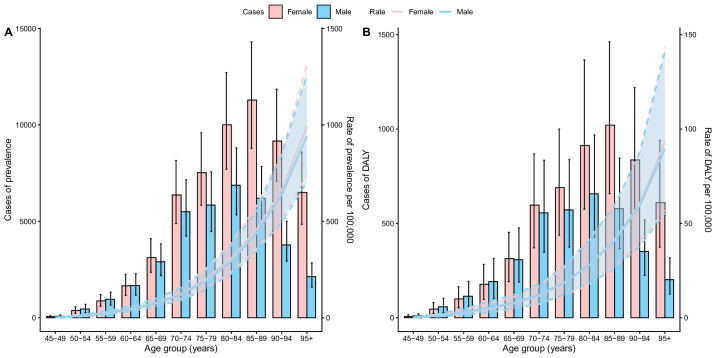
(**A, B**) Rate per 100,000 of the population of prevalence (**A**) and DALY rate (**B**) from vision impairment due to AMD in Japan by age group and sex in 2021. *Shaded regions* indicate 95% UIs.

In terms of age distribution, both the number of prevalent cases and DALYs in 2021 exhibited an inverted U-shaped curve, peaking in the 80 to 84 age group, whereas the prevalence and DALY rates increased continuously with age (Fig. 2). From 1990 to 2021, all age groups experienced consistent declines in prevalence and DALY rates, with more pronounced reductions observed in younger groups. The 45 to 49 age group showed a PC of −17.35% (95% UI, −28.02 to −4.12) in prevalence rate and −24.76% (95% UI, −35.42 to −12.53) in DALY rate. In contrast, the ≥95 age group exhibited smaller declines, with PCs of −6.96% (95% UI, −9.06 to −4.70) in prevalence rate and −9.43% (95% UI, −13.10 to −5.19) in DALY rate (Table; Supplementary Tables S1, S4, S5).

### Moderate to Severe Vision Loss and Blindness due to AMD

**Figure 3.**
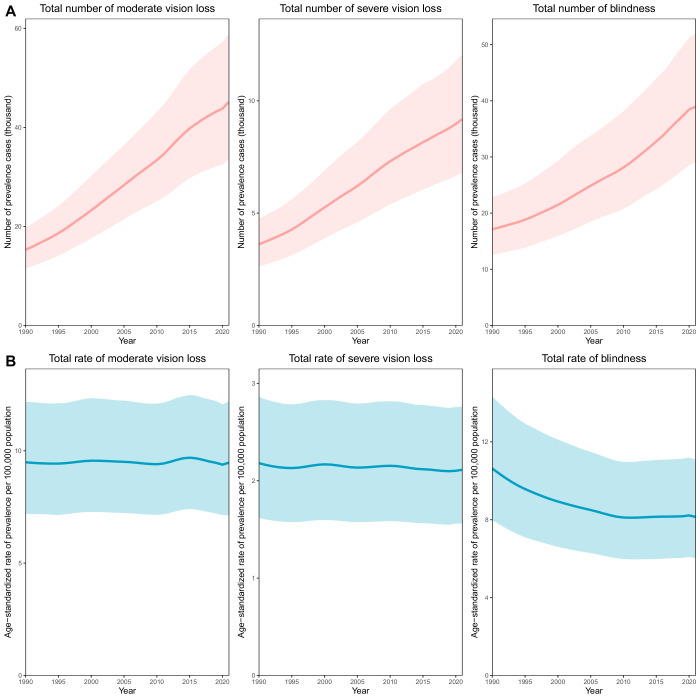
Vision impairment due to AMD by constituent sequelae prevalence for both sexes combined from 1990 to 2021. (**A**) Total number of moderate vision loss, severe vision loss, and blindness. (**B**) Rates per 100,000 population of age-standardized moderate vision loss, severe vision loss, and blindness. *Shaded regions* indicate 95% UIs.

From 1990 to 2021, the total prevalence of vision impairment due to AMD—categorized as moderate vision loss, severe vision loss, and blindness—showed an increase in the number of cases, alongside a slight decline in age-standardized prevalence rates. The number of cases of moderate vision loss increased from 15,288 (95% UI, 11,537–19,753) to 45,186 (95% UI, 33,668–58,849), whereas its age-standardized rate remained largely stable, changing minimally from 9.49 (95% UI, 7.20–12.19) to 9.47 (95% UI, 7.13–12.20) per 100,000 population. Cases of severe vision loss rose from 3617 (95% UI, 2635–4756) to 9186 (95% UI, 6801–12,056), with a slight decrease in the age-standardized rate from 2.18 (95% UI, 1.62–2.86) to 2.11 (95% UI, 1.56–2.76). The number of blindness cases increased from 17,122 (95% UI, 12,588–22,831) to 38,938 (95% UI, 28,979–51,898), but the age-standardized rate declined from 10.63 (95% UI, 7.98–14.32) to 8.14 (95% UI, 6.02–11.09) per 100,000 (Fig. 3; Supplementary Tables S6, S8).

In terms of disease burden, the number of DALYs due to moderate vision loss increased from 463 (95% UI, 261–775) to 1363 (95% UI, 771–2240), with the corresponding rate remaining stable at approximately 0.29 (95% UI, 0.16–0.48) per 100,000. DALYs attributable to severe vision loss rose from 647 (95% UI, 410–1002) to 1631 (95% UI, 1022–2541), whereas the rate slightly decreased from 0.39 (95% UI, 0.25–0.60) to 0.38 (95% UI, 0.24–0.59). The largest decline was observed in blindness-related DALYs, which increased in number from 3114 (95% UI, 1764–4683) to 7009 (95% UI, 4198–10,383), but decreased in rate from 1.93 (95% UI, 1.10–2.84) to 1.48 (95% UI, 0.84–2.18) per 100,000 (Supplementary Fig. S1; Supplementary Tables S7, S9).

### Burden of Vision Impairment Due to AMD Attributable to Tobacco

**Figure 4.**
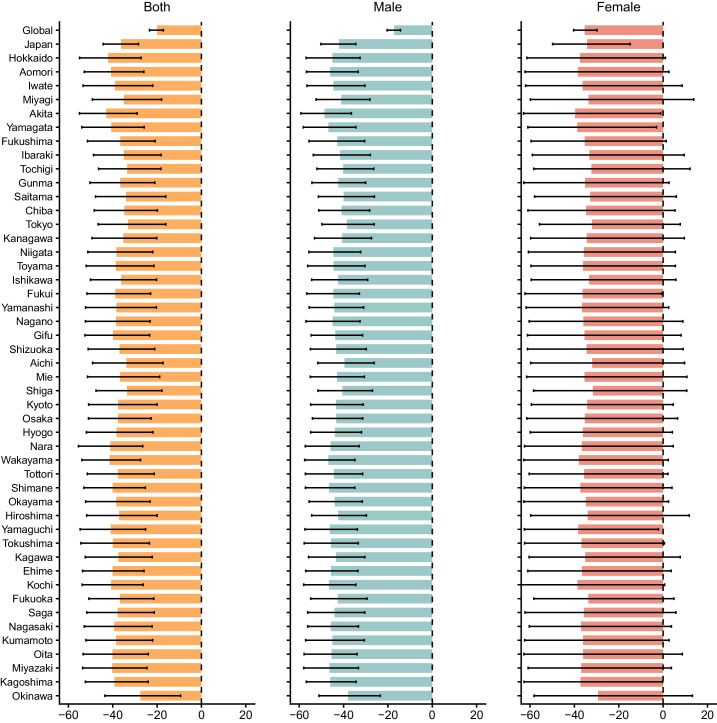
Percentage change in age-standardized DALY rates attributed to tobacco as a risk factor from vision impairment due to AMD by prefectures from 1990 to 2021. *Error bars* indicate 95% UIs.

The age-standardized DALY rate for vision impairment due to AMD attributable to tobacco showed a continuous decline, decreasing from 0.36 (95% UI, 0.19–0.57) per 100,000 population in 1990 to 0.19 (95% UI, 0.10–0.32) in 2021, with a PC of −47.23 (95% UI, −54.67 to −39.89). The reduction was 51.35% (PC, −58.81 to −43.93) in males and was 46.36% (PC, −59.65 to −30.69) in females. In 2021, the age-standardized DALY rates across prefectures ranged between 0.17 and 0.22, indicating minimal regional variation. Concurrently, the proportion of tobacco-attributable DALYs among total AMD-related DALYs decreased from 15.19% (95% UI, 9.41–21.25) in 1990 to 9.68% (95% UI, 5.57–14.34) in 2021, reflecting an overall decline of 36.3% (95% UI, −44.33 to −28.36). The proportion in males decreased from 28.25% (95% UI, 17.98–38.57) to 16.35% (95% UI, 9.71–23.99), a reduction of 42.12% (95% UI, −50.25 to −34.48), whereas in females it declined from 6.15% (95% UI, 3.38–9.37) to 4.04% (95% UI, 2.13–6.32), a decrease of 34.29% (95% UI, −49.81 to −14.86). Reductions across prefectures ranged from −27% to −43% (Fig. 4; Supplementary Tables S10, S11).

### Burden Projections to 2040

Projections based on the ARIMA model indicate distinct future trajectories for AMD-related vision impairment by sex. For the overall population, the total number of cases is projected to increase by 42.51% by 2040 compared to 2021 levels, reaching 132,976 cases (95% confidence interval [CI], 120,258–145,693). However, sex-stratified projections reveal divergent trends in age-standardized prevalence rates: The male rate is projected to increase to 20.6 per 100,000 (95% CI, 17.1–25.3), but the female rate is projected to decline to 14.29 per 100,000 (95% CI, 5.7–22.9). The underlying ARIMA(0,2,1) model parameters help explain this divergence: The estimated MA(1) coefficient was significantly negative for males (–0.538, *P* = 0.013) but non-significantly positive for females (+0.583, *P* = 0.225), suggesting a potential inflection point in the male trend.

## Discussion

This study systematically evaluated the burden and trends of vision impairment due to AMD in Japan from 1990 to 2021. The results indicate that both the age-standardized prevalence rate and age-standardized DALY rate of AMD-related vision impairment showed a declining trend. Demographic disparities were observed, with a slightly higher prevalence among females compared to males. Both the prevalence and DALY rates increased with age, with individuals aged 70 years and above constituting the most affected population. Furthermore, a consistent decrease in the age-standardized DALY rate was observed from 1990 to 2021. This reduction may be partially attributable to a 36.3% decline in DALYs related to tobacco use over the same period.

From 1990 to 2021, Japan witnessed a significant decline in both the age-standardized prevalence rate and age-standardized DALY rate of vision impairment due to AMD, reflecting progress in AMD prevention and control over the past three decades. This trend aligns with the overall global reduction in AMD-related vision impairment burden^4^^,^^15^; however, the annual rate of decline in Japan exceeded the global average, particularly in terms of DALYs. This discrepancy may be attributed to Japan's early transition to a super-aged society coupled with the concurrent implementation of nationwide eye health policies. The global decline in AMD-related vision impairment has been largely driven by high-income countries, with high-income Asia–Pacific countries, including Japan, demonstrating steeper reductions due to enhanced accessibility to anti-vascular endothelial growth factor (VEGF) therapies and improved primary-level screening programs.^13^ Although the absolute number of cases increased due to population aging, the sustained decline in age-standardized rates suggests that the expansion of AMD has been slower than the pace of demographic aging, indicating relative success in the management of age-related eye diseases in Japan.

Sex and age stratification revealed a slightly higher AMD-related vision impairment prevalence among females compared to males. This disparity may be attributed to the regulatory role of estrogen metabolic pathways in the pathological processes of AMD.^16^ However, the difference in age-standardized DALY rates between males and females was minimal. In contrast, global studies often report a significantly higher disease burden in females.^13^ The observed convergence in disease burden between sexes in Japan may be associated with narrowed disparities in healthcare utilization and timeliness of anti-VEGF treatment.^17^ Furthermore, the disease burden increased significantly with age, with adults 70 years of age and above bearing the majority of the burden. This pattern is consistent with the age and sex distribution characteristics of AMD reported in most developed countries, underscoring that the elderly population should be the primary target for AMD prevention and management strategies.^3^

Among Japan's 47 prefectures, the burden of vision impairment due to AMD exhibited a high degree of homogeneity. All regions demonstrated significant declines in age-standardized rates. In 2021, inter-prefecture variation was minimal, with prevalence ranging between 16.58 and 24.98 per 100,000 population, and DALY rates clustering within a narrow range (1.89–2.09 per 100,000). This pattern reflects considerable equity in healthcare resource allocation and public health service delivery across Japan, standing in sharp contrast to the significantly higher burden typically observed in low-income regions in global studies.^13^ This homogeneity may be attributed to the equitable distribution of medical resources under Japan's universal health coverage system. The nationwide unified health insurance scheme and standardized clinical practices have likely mitigated the impact of regional socioeconomic disparities on health outcomes.^17^ In terms of diagnosis, optical coherence tomography (OCT) has been widely adopted since its inclusion in public health insurance coverage in 2008, becoming a vital tool for early screening and diagnosis of AMD and facilitating timely detection and management.^17^ Regarding treatment, although anti-VEGF therapy is associated with high costs, its inclusion in insurance coverage since 2008, with patient co-payments as low as 10% to 30%, has resulted in substantially higher accessibility in Japan compared to many other countries.^13^^,^^17^^,^^18^ This has considerably reduced the risk of vision impairment due to AMD. Furthermore, Japan has increasingly strengthened public health education and professional physician training related to AMD in recent years, enhancing public awareness and compliance with early treatment.^17^ Collectively, these factors may have contributed to the observed decline in age-standardized DALY rates.

Aging is the strongest non-modifiable risk factor for AMD.^2^ Among modifiable risk factors, smoking is the most well established, with previous studies indicating that it can increase the risk of developing AMD by up to threefold.^1^ In Japan, the reduction in tobacco use has likely played an important role in the decline of the age-standardized DALY rate. From 1990 to 2021, the proportion of AMD-related vision impairment DALYs attributable to tobacco use decreased from 15.19% to 9.68%, a reduction of 36.3%. The decline was more pronounced in males (42.12%) than in females (34.29%), and both exceeded the global average decline of 20.03%. This trend may be closely linked to Japan's progressively strengthened tobacco control policies over recent decades, including increases in tobacco taxes, legislative restrictions on smoking in public places, and gender-specific tobacco control strategies.^19^^–^^21^ Japan approved the World Health Organization Framework Convention on Tobacco Control (FCTC) over two decades ago, and its nationwide tobacco control measures have shown early success. The adult smoking rate declined significantly from 24.9% in 2005 to 14.8% in 2022.^22^^,^^23^ Moreover, as early smoking cessation interventions were primarily targeted at men, the decline in smoking prevalence has been more substantial among males, accompanied by higher quit rates.^24^ This disparity underscores the importance of gender-specific public health strategies in effectively reducing smoking rates and highlights the need to enhance the accessibility of cessation support across different populations.^25^^–^^27^ Consistent evidence indicates that strengthened tobacco control, including fiscal policies and public health education, can further reduce the burden of smoking-related diseases.^28^ Against the backdrop of a rapidly aging society, the rising number of AMD-related vision impairment cases continues to impose significant economic pressure on the healthcare system due to increased medical expenditures.^17^^,^^29^^,^^30^ Therefore, developing more cost-effective prevention and intervention strategies is essential for controlling the disease burden of AMD-related vision impairment. In this context, reducing tobacco use, as a well-established and modifiable risk factor, may represent a critical strategy for preventing AMD and associated vision impairment.

The ARIMA model projections revealed a distinct sex-specific pattern: Although the age-standardized rate of AMD-related vision impairment burden is projected to continue declining among females, it shows signs of stabilization or a slight increase among males (Supplementary Fig. S2; Supplementary Table S13). This divergence in forecast trajectories can be explained by both statistical characteristics of the data and underlying epidemiological factors. The selected ARIMA model for males, with a significant negative MA(1) coefficient of –0.538 (*P* = 0.013), captured a subtle “dip-and-rebound” pattern in the recent data (2019–2021), which the model interprets as a potential inflection point in the previously declining trend. From a public health perspective, this projected plateau or rise may reflect the persistent accumulation of AMD-related vision impairment risk among older male cohorts due to historically high smoking prevalence. Although overall smoking rates in Japan have declined, male smoking rates remain substantially higher than female rates, and the pace of decline may have slowed in recent years. Thus, against the backdrop of a rapidly aging population, the lagged effect of past smoking exposure, coupled with possible gendered differences in the effectiveness of tobacco control measures, could lead to a rebound in smoking-attributable AMD-related vision impairment burden among males. This finding underscores the need for continued surveillance and the importance of strengthening targeted, sex-specific tobacco control policies to mitigate future vision impairment.

This study has several limitations inherent to its data sources and analytical methods. First, although the GBD database employs standardized estimation techniques, its reliance on modeled estimates synthesized from multiple, heterogeneous data sources may not fully capture prefectural heterogeneity within Japan. Inconsistencies in AMD diagnosis and reporting practices, particularly at the primary healthcare level, could affect the accuracy of the estimates. Second, for the risk factor attribution analysis, behavioral data on tobacco use primarily derive from population surveys and statistical modeling, which are susceptible to measurement error and recall bias. The analysis did not adjust for all potential individual-level confounders (specific comorbidities, genetic factors, or detailed lifestyle measures), which may affect the precise quantification of the contribution of tobacco to the AMD-related vision impairment burden. This limitation is particularly relevant when interpreting estimates for subgroups such as female, where lower smoking prevalence and greater data heterogeneity can result in wider uncertainty intervals. Third, although the widespread adoption of OCT and utilization of anti-VEGF therapy are considered key drivers in reducing AMD-related vision impairment burden, this study did not directly evaluate their specific impacts or apportion their contributions to the observed decline. Fourth, the ARIMA model used for forecasting, although effective at capturing historical linear trends and autocorrelation, is inherently an extrapolation of past patterns. It does not actively incorporate potential future disruptions, such as major public health interventions, emerging epidemics, or breakthroughs in medical technology. Consequently, long-term projections are subject to uncertainty. Finally, due to the consistent application of GBD methodology, estimates for certain subgroups or regions are associated with wide uncertainty intervals, which may constrain the interpretation of subtle differences and temporal trends. Despite these limitations, the strong established evidence supporting tobacco as a major AMD risk factor underscores the validity of the observed trends. This study provides valuable macro-level insights into the long-term characteristics of AMD-related vision impairment burden in Japan and offers meaningful information to guide public health decision-making.

## Conclusions

From 1990 to 2021, Japan experienced an increase in the number of AMD-related vision impairment cases and DALYs; yet, both the age-standardized prevalence rate and age-standardized DALY rate declined, with the most pronounced reduction observed in the burden of blindness due to AMD. The broad accessibility of anti-VEGF therapy and OCT screening under universal health insurance coverage, coupled with gender-specific tobacco control policies, likely played a critical role in reducing the burden of vision impairment attributable to AMD. Future efforts should focus on promoting cost-effective diagnostic and treatment strategies, strengthening tobacco control measures, and implementing comprehensive interventions targeting modifiable risk factors.

## Supplementary Material

Supplement 1

## References

[1] Guymer RH, Campbell TG. Age-related macular degeneration. *Lancet*. 2023; 401(10386): 1459–1472. 36996856 10.1016/S0140-6736(22)02609-5

[2] Apte RS. Age-related macular degeneration. *N Engl J Med*. 2021; 385(6): 539–547. 34347954 10.1056/NEJMcp2102061 PMC9369215

[3] Wong WL, Su X, Li X, et al. Global prevalence of age-related macular degeneration and disease burden projection for 2020 and 2040: a systematic review and meta-analysis. *Lancet Glob Health*. 2014; 2(2): e106–e116. 25104651 10.1016/S2214-109X(13)70145-1

[4] Vision Loss Expert Group of the Global Burden of Disease Study; GBD 2019 Blindness and Vision Impairment Collaborators. Global estimates on the number of people blind or visually impaired by age-related macular degeneration: a meta-analysis from 2000 to 2020. *Eye (Lond)*. 2024; 38(11): 2070–2082. 38965321 10.1038/s41433-024-03050-z PMC11269688

[5] GBD 2019 Blindness and Vision Impairment Collaborators; Vision Loss Expert Group of the Global Burden of Disease Study. Trends in prevalence of blindness and distance and near vision impairment over 30 years: an analysis for the Global Burden of Disease Study. *Lancet Glob Health*. 2021; 9(2): e130–e143. 33275950 10.1016/S2214-109X(20)30425-3 PMC7820390

[6] Seddon JM, Cote J, Rosner B. Progression of age-related macular degeneration: association with dietary fat, transunsaturated fat, nuts, and fish intake. *Arch Ophthalmol*. 2003; 121(12): 1728–1737. 14662593 10.1001/archopht.121.12.1728 PMC8443211

[7] Rim TH, Cheng CY, Kim DW, Kim SS, Wong TY. A nationwide cohort study of cigarette smoking and risk of neovascular age-related macular degeneration in East Asian men. *Br J Ophthalmol*. 2017; 101(10): 1367–1373. 28292774 10.1136/bjophthalmol-2016-309952

[8] Chakravarthy U, Augood C, Bentham GC, et al. Cigarette smoking and age-related macular degeneration in the EUREYE Study. *Ophthalmology*. 2007; 114(6): 1157–1163. 17337063 10.1016/j.ophtha.2006.09.022

[9] Chen Z, Peto R, Zhou M, et al. Contrasting male and female trends in tobacco-attributed mortality in China: evidence from successive nationwide prospective cohort studies. *Lancet*. 2015; 386(10002): 1447–1456. 26466050 10.1016/S0140-6736(15)00340-2 PMC4691901

[10] Nakata I, Yamashiro K, Nakanishi H, et al. Prevalence and characteristics of age-related macular degeneration in the Japanese population: the Nagahama study. *Am J Ophthalmol*. 2013; 156(5): 1002–1009.e2. 23938127 10.1016/j.ajo.2013.06.007

[11] Sobrin L, Seddon JM. Nature and nurture– genes and environment– predict onset and progression of macular degeneration. *Prog Retin Eye Res*. 2014; 40: 1–15. 24374240 10.1016/j.preteyeres.2013.12.004 PMC6446565

[12] GBD 2021 Diseases and Injuries Collaborators. Global incidence, prevalence, years lived with disability (YLDs), disability-adjusted life-years (DALYs), and healthy life expectancy (HALE) for 371 diseases and injuries in 204 countries and territories and 811 subnational locations, 1990–2021: a systematic analysis for the Global Burden of Disease Study 2021. *Lancet*. 2024; 403(10440): 2133–2161. 38642570 10.1016/S0140-6736(24)00757-8 PMC11122111

[13] GBD 2021 Global AMD Collaborators. Global burden of vision impairment due to age-related macular degeneration, 1990–2021, with forecasts to 2050: a systematic analysis for the Global Burden of Disease Study 2021. *Lancet Glob Health*. 2025; 13(7): e1175–e1190. 40580986 10.1016/S2214-109X(25)00143-3 PMC12208786

[14] GBD 2021 Risk Factor Collaborators. Global burden and strength of evidence for 88 risk factors in 204 countries and 811 subnational locations, 1990-2021: a systematic analysis for the Global Burden of Disease Study 2021. *Lancet*. 2024; 403(10440): 2162–2203. 38762324 10.1016/S0140-6736(24)00933-4 PMC11120204

[15] Hu P, He M, Cai J, et al. Global burden of smoking-associated age-related macular degeneration: spatiotemporal trends from 1990 to 2021 and projections to 2040. *Tob Induc Dis*. 2025; 23. 10.18332/tid/205665 PMC12247051 40656874

[16] Hwang S, Kang SW, Han J, et al. Female reproductive factors and the risk of exudative age-related macular degeneration: a nationwide cohort study. *Retina*. 2021; 41(10): 2088–2097. 33675332 10.1097/IAE.0000000000003164

[17] Kume A, Ohshiro T, Sakurada Y, Kikushima W, Yoneyama S, Kashiwagi K. Treatment patterns and health care costs for age-related macular degeneration in Japan: an analysis of national insurance claims data. *Ophthalmology*. 2016; 123(6): 1263–1268. 26927204 10.1016/j.ophtha.2016.01.042

[18] Kido A, Miyake M, Tamura H, et al. Incidence and clinical practice of exudative age-related macular degeneration: a nationwide population-based cohort study. *Ophthalmol Sci*. 2022; 2(2): 100125. 36249688 10.1016/j.xops.2022.100125 PMC9559904

[19] Tabuchi T, Nakamura M, Nakayama T, Miyashiro I, Mori J, Tsukuma H. Tobacco price increase and smoking cessation in Japan, a developed country with affordable tobacco: a national population-based observational study. *J Epidemiol*. 2016; 26(1): 14–21. 26277880 10.2188/jea.JE20140183 PMC4690736

[20] Tanaka S, Ihira H, Tajima T, et al. Birth cohort-specific smoking patterns in Japan (1906–2004): a population-based study from the NC-CCAPH consortium. *Lancet Reg Health West Pac*. 2025; 58: 101562. 40475886 10.1016/j.lanwpc.2025.101562 PMC12138583

[21] Nguyen PT, Tanaka S, Fukui K, Ito Y, Katanoda K. Patterns of birth cohort-specific smoking histories in Japan, 1910–2050: a shift towards younger people? [published online ahead of print July 1, 2025]. *Tob Control*. 2025. 10.1136/tc-2024-059262. 40592573

[22] Sternbach N, Annunziata K, Fukuda T, Yirong C, Stankus AP. Smoking trends in Japan from 2008-2017: results from the National Health and Wellness Survey. *Value Health*. 2018; 21: S105.

[23] Ministry of Health, Labour and Welfare. Overview of the results from the 2022 National Health and Nutrition Survey in Japan. Available at: https://www.mhlw.go.jp/content/10900000/001338334.pdf. Accessed February 6, 2026.

[24] Li M, Okamoto R. Gender differences and smoking cessation in the Japanese smoking cessation treatment program. *Tob Induc Dis*. 2020; 18: 95. 33223985 10.18332/tid/128497 PMC7676305

[25] Nguyen PT, Rahman MS, Le PM, et al. Trends in, projections of, and inequalities in reproductive, maternal, newborn and child health service coverage in Vietnam 2000–2030: a Bayesian analysis at national and sub-national levels. *Lancet Reg Health West Pac*. 2021; 15: 100230. 34528011 10.1016/j.lanwpc.2021.100230 PMC8342952

[26] Nguyen PT, Gilmour S, Le PM, et al. Trends in, projections of, and inequalities in non-communicable disease management indicators in Vietnam 2010–2030 and progress toward universal health coverage: a Bayesian analysis at national and sub-national levels. *EClinicalMedicine*. 2022; 51: 101550. 35856038 10.1016/j.eclinm.2022.101550 PMC9287489

[27] Smith PH, Kasza KA, Hyland A, et al. Gender differences in medication use and cigarette smoking cessation: results from the International Tobacco Control Four Country Survey. *Nicotine Tob Res*. 2015; 17(4): 463–472. 25762757 10.1093/ntr/ntu212 PMC4402353

[28] Bialous S, Da Costa ESVL. Where next for the WHO Framework Convention on Tobacco Control? *Tob Control*. 2022; 31(2): 183–186. 35241586 10.1136/tobaccocontrol-2021-056545

[29] Huang L, Hu P, Chen J, Liu J, Grzybowski A. Smoking-attributable age-related macular degeneration vision loss in China: 1990–2021 trends and 2040 projections. *Transl Vis Sci Technol*. 2025; 14(10): 21. 10.1167/tvst.14.10.21 PMC12534889 41099595

[30] Chen YH, Li Y, Hu PC, Chen T, Grzybowski A, Huang LM. Global burden of vision impairment due to smoking-related cataract: a descriptive study of spatiotemporal trends based on GBD secondary data and projections to 2050. *Tob Induc Dis*. 2025; 23. 10.18332/tid/210411 PMC12579110 41180462

