# Supplementary material for: Tobacco-Attributable Age-Related Macular Degeneration Vision Impairment in Japan: A National and Prefecture-Level Analysis From 1990 to 2040
Source: Transl Vis Sci Technol. 2026 Feb 18;15(2):23. doi: 10.1167/tvst.15.2.23 (PMC12924141; doi:10.1167/tvst.15.2.23)
Supplement: Supplement 1 [file tvst-15-2-23_s001.docx]

| Table S1. Cases and percentage change of prevalence and DALYs of vision impairment due to age-related macular degeneration by prefectures from 1990 to 2021 | | | | | | |
| --- | --- | --- | --- | --- | --- | --- |
|  | Prevalence | | | DALYs | | |
|  | 1990 (95% UI) | 2021 (95% UI) | Percentage change between 1990 and 2021, % (95%UI) | 1990 (95% UI) | 2021 (95% UI) | Percentage change between 1990 and 2021, % (95%UI) |
| **Global** | 3640180 (3037098 to 4353902) | 8057521 (6705284 to 9823238) | 121.35 (114.01 to 128.68) | 302902 (206475 to 421952) | 578020 (401241 to 797570) | 90.83 (82.13 to 100.23) |
| **Japan** | 36027 (29884 to 43516) | 93310 (78103 to 112033) | 159 (138.06 to 182.66) | 3814 (2442 to 5349) | 8907 (5984 to 12298) | 133.53 (110.4 to 163.02) |
| **Sex** |  |  |  |  |  |  |
| Male | 14127 (11580 to 17266) | 36375 (30344 to 44085) | 157.48 (137.44 to 183.12) | 1549 (991 to 2181) | 3597 (2407 to 5005) | 132.31 (106.91 to 163.08) |
| Female | 21899 (18256 to 26150) | 56935 (47768 to 68506) | 159.98 (137.42 to 187.1) | 2265 (1469 to 3200) | 5309 (3575 to 7403) | 134.36 (108.75 to 165.45) |
| **Age** |  |  |  |  |  |  |
| 45-49 years | 155 (74 to 283) | 138 (69 to 248) | -10.88 (-22.39 to 3.38) | 21 (8 to 45) | 17 (7 to 37) | -18.88 (-30.37 to -5.68) |
| 50-54 years | 891 (524 to 1363) | 832 (490 to 1267) | -6.58 (-14.41 to 3.5) | 122 (58 to 212) | 104 (50 to 182) | -14.99 (-22.39 to -6.4) |
| 55-59 years | 2173 (1471 to 3064) | 1829 (1256 to 2515) | -15.8 (-20.91 to -9.73) | 274 (148 to 457) | 214 (114 to 346) | -21.86 (-36.5 to -2.98) |
| 60-64 years | 3545 (2435 to 4866) | 3326 (2340 to 4520) | -6.19 (-11.09 to -0.42) | 424 (234 to 682) | 369 (200 to 588) | -13.03 (-24.72 to 0.34) |
| 65-69 years | 4465 (3347 to 5972) | 6022 (4546 to 7959) | 34.87 (28.91 to 41.28) | 497 (292 to 748) | 621 (382 to 921) | 24.93 (9.54 to 40.57) |
| 70-74 years | 5299 (4072 to 6842) | 11865 (9118 to 15245) | 123.92 (114.8 to 132.69) | 553 (345 to 835) | 1153 (710 to 1695) | 108.38 (87.2 to 134.06) |
| 75-79 years | 6723 (5202 to 8671) | 13367 (10292 to 17174) | 98.83 (90.9 to 105.04) | 677 (440 to 991) | 1261 (818 to 1826) | 86.24 (67.71 to 107.76) |
| 80-84 years | 6206 (4800 to 7971) | 16879 (13064 to 21486) | 171.97 (162.77 to 180.04) | 614 (392 to 918) | 1570 (1013 to 2311) | 155.5 (137.58 to 175.48) |
| 85-89 years | 4173 (3275 to 5334) | 17483 (13600 to 22155) | 318.95 (307.09 to 331.28) | 402 (253 to 585) | 1598 (1040 to 2311) | 297.91 (275.86 to 321.44) |
| 90-94 years | 1846 (1445 to 2391) | 12939 (10000 to 16827) | 600.86 (584.1 to 619.47) | 176 (112 to 258) | 1188 (782 to 1732) | 574.51 (539.09 to 612.07) |
| 95+ years | 550 (407 to 730) | 8628 (6447 to 11382) | 1467.52 (1432.18 to 1505.65) | 53 (33 to 82) | 812 (497 to 1260) | 1425.86 (1364.04 to 1497.4) |
| **Prefectures** |  |  |  |  |  |  |
| Hokkaido | 1611 (1333 to 1963) | 4347 (3631 to 5268) | 169.82 (143.17 to 201.59) | 173 (111 to 244) | 415 (278 to 575) | 140.53 (98.55 to 193.12) |
| Aomori | 475 (391 to 577) | 1090 (907 to 1314) | 129.61 (106.14 to 153.96) | 51 (33 to 72) | 104 (68 to 145) | 104.5 (70.19 to 145.37) |
| Iwate | 497 (409 to 601) | 1100 (917 to 1314) | 121.46 (100.26 to 145.77) | 53 (34 to 75) | 105 (69 to 146) | 97.74 (62.76 to 144.18) |
| Miyagi | 627 (521 to 758) | 1700 (1438 to 2034) | 171.07 (145.06 to 199.97) | 67 (41 to 96) | 161 (110 to 225) | 142.35 (102.18 to 199.39) |
| Akita | 449 (371 to 542) | 971 (812 to 1169) | 116.11 (93.04 to 141.57) | 48 (31 to 68) | 93 (62 to 129) | 93.78 (61.07 to 136.56) |
| Yamagata | 488 (406 to 592) | 1008 (845 to 1204) | 106.79 (86.07 to 132.13) | 51 (33 to 74) | 96 (63 to 134) | 86.35 (53.97 to 125.15) |
| Fukushima | 724 (598 to 878) | 1546 (1296 to 1856) | 113.43 (93.98 to 137.84) | 77 (50 to 111) | 147 (102 to 207) | 90.99 (58.61 to 131.14) |
| Ibaraki | 805 (670 to 971) | 2144 (1809 to 2573) | 166.5 (142.16 to 194.13) | 86 (54 to 123) | 205 (136 to 284) | 138.82 (98.83 to 186.83) |
| Tochigi | 564 (471 to 677) | 1423 (1191 to 1716) | 152.13 (130.53 to 177.74) | 60 (37 to 86) | 137 (90 to 195) | 128.02 (91.43 to 170.08) |
| Gunma | 609 (506 to 739) | 1506 (1249 to 1810) | 147.35 (123.72 to 173.95) | 64 (40 to 90) | 143 (93 to 197) | 123.22 (86.31 to 168.21) |
| Saitama | 1296 (1067 to 1574) | 4839 (4064 to 5844) | 273.29 (237.32 to 312.68) | 139 (87 to 201) | 462 (307 to 648) | 233.21 (179.38 to 304.57) |
| Chiba | 1246 (1033 to 1515) | 4305 (3562 to 5202) | 245.63 (215.55 to 280.5) | 133 (85 to 192) | 410 (274 to 566) | 209.18 (158.68 to 281.03) |
| Tokyo | 2990 (2463 to 3603) | 8386 (6982 to 10109) | 180.42 (153.03 to 211.83) | 314 (200 to 447) | 795 (542 to 1102) | 152.95 (111.84 to 201.01) |
| Kanagawa | 1703 (1403 to 2064) | 6021 (4998 to 7255) | 253.65 (220.64 to 294.09) | 180 (115 to 262) | 576 (381 to 803) | 219.28 (165.96 to 289.55) |
| Niigata | 882 (731 to 1070) | 1908 (1586 to 2301) | 116.24 (95.68 to 139.59) | 92 (60 to 132) | 182 (122 to 257) | 96.7 (64.8 to 135.96) |
| Toyama | 408 (339 to 494) | 900 (756 to 1073) | 120.44 (100.28 to 144.82) | 43 (28 to 62) | 86 (58 to 122) | 99.5 (68.41 to 138.37) |
| Ishikawa | 386 (321 to 470) | 888 (747 to 1065) | 129.81 (109.59 to 153.59) | 41 (25 to 57) | 84 (56 to 116) | 108.36 (73.14 to 148.31) |
| Fukui | 301 (250 to 361) | 640 (538 to 767) | 112.57 (92.69 to 136.3) | 32 (20 to 45) | 61 (41 to 85) | 92.56 (61.55 to 131.51) |
| Yamanashi | 303 (250 to 364) | 670 (561 to 808) | 121.48 (102.58 to 144.89) | 32 (21 to 45) | 64 (42 to 89) | 101.15 (63.15 to 138.63) |
| Nagano | 816 (675 to 977) | 1796 (1496 to 2161) | 120.09 (100.01 to 146.63) | 85 (54 to 121) | 170 (113 to 240) | 100.08 (67.63 to 141.43) |
| Gifu | 600 (496 to 731) | 1563 (1304 to 1864) | 160.39 (133.94 to 186.96) | 62 (39 to 89) | 148 (99 to 203) | 139.25 (100.16 to 187.42) |
| Shizuoka | 1079 (891 to 1307) | 2833 (2346 to 3407) | 162.47 (140.55 to 191.75) | 114 (74 to 166) | 268 (176 to 377) | 135.55 (98.84 to 183.96) |
| Aichi | 1502 (1213 to 1845) | 4549 (3747 to 5573) | 202.92 (172.92 to 235.19) | 167 (103 to 239) | 451 (293 to 636) | 170.72 (126.39 to 231.21) |
| Mie | 598 (498 to 724) | 1403 (1180 to 1689) | 134.51 (113.68 to 158.84) | 63 (40 to 91) | 133 (89 to 189) | 111.39 (74.71 to 156.61) |
| Shiga | 352 (291 to 428) | 945 (787 to 1135) | 168.09 (144.71 to 195.41) | 37 (23 to 53) | 90 (60 to 124) | 144.04 (103.35 to 196.71) |
| Kyoto | 823 (681 to 999) | 1976 (1653 to 2362) | 140.17 (121.38 to 161.03) | 86 (55 to 123) | 188 (125 to 260) | 117.52 (85.66 to 162.03) |
| Osaka | 2091 (1720 to 2529) | 6184 (5142 to 7436) | 195.78 (166.87 to 231.13) | 224 (143 to 320) | 593 (395 to 827) | 164.48 (120.69 to 222.37) |
| Hyogo | 1572 (1301 to 1902) | 4094 (3389 to 4907) | 160.52 (137.27 to 187.75) | 166 (106 to 236) | 390 (254 to 537) | 134.11 (95.06 to 184.16) |
| Nara | 386 (320 to 464) | 1072 (900 to 1286) | 177.57 (153.68 to 203.54) | 41 (26 to 58) | 102 (69 to 143) | 149.85 (110.84 to 200.03) |
| Wakayama | 400 (329 to 485) | 802 (671 to 957) | 100.56 (82.66 to 121.87) | 42 (27 to 61) | 76 (51 to 107) | 81.57 (51.7 to 116.61) |
| Tottori | 240 (200 to 289) | 479 (403 to 580) | 99.55 (81.24 to 120.28) | 25 (16 to 36) | 46 (31 to 64) | 81.92 (51.71 to 121.73) |
| Shimane | 357 (299 to 430) | 662 (555 to 796) | 85.34 (67.9 to 105.32) | 37 (24 to 53) | 62 (42 to 88) | 67.48 (43.93 to 99.66) |
| Okayama | 713 (590 to 866) | 1538 (1289 to 1858) | 115.74 (97.35 to 134.88) | 74 (47 to 105) | 146 (97 to 205) | 96.57 (65.11 to 136.41) |
| Hiroshima | 921 (765 to 1120) | 2161 (1805 to 2585) | 134.69 (114.34 to 159.32) | 96 (62 to 137) | 205 (139 to 291) | 112.97 (82.48 to 156.24) |
| Yamaguchi | 606 (503 to 735) | 1229 (1025 to 1474) | 102.65 (84.9 to 125.53) | 64 (41 to 91) | 117 (77 to 163) | 83.13 (53.79 to 123.26) |
| Tokushima | 318 (265 to 384) | 658 (549 to 797) | 107.37 (88.62 to 130.05) | 33 (22 to 47) | 63 (42 to 88) | 88.21 (57.9 to 126.23) |
| Kagawa | 386 (321 to 465) | 816 (687 to 978) | 111.56 (91.8 to 133.46) | 40 (25 to 57) | 78 (51 to 107) | 93.04 (63.85 to 129.11) |
| Ehime | 572 (476 to 691) | 1201 (1004 to 1444) | 109.91 (90.26 to 131.45) | 60 (39 to 86) | 114 (74 to 161) | 89.35 (58.05 to 125.03) |
| Kochi | 354 (296 to 425) | 678 (565 to 811) | 91.7 (72.21 to 111.53) | 37 (24 to 53) | 65 (43 to 91) | 73.24 (45.73 to 104.77) |
| Fukuoka | 1464 (1204 to 1770) | 3682 (3072 to 4423) | 151.49 (130.3 to 176.32) | 154 (97 to 223) | 349 (234 to 487) | 126.08 (86.25 to 171.55) |
| Saga | 334 (277 to 403) | 673 (561 to 806) | 101.67 (84.09 to 121.87) | 35 (23 to 50) | 64 (42 to 91) | 83.19 (54.74 to 114.08) |
| Nagasaki | 558 (463 to 676) | 1164 (980 to 1384) | 108.76 (90.06 to 130.92) | 59 (38 to 85) | 111 (74 to 155) | 87.91 (57.87 to 125.06) |
| Kumamoto | 703 (584 to 848) | 1500 (1248 to 1812) | 113.53 (94.77 to 136) | 74 (48 to 103) | 143 (97 to 199) | 93.82 (65.07 to 131.48) |
| Oita | 464 (381 to 561) | 1005 (836 to 1200) | 116.59 (96.65 to 140.58) | 49 (31 to 70) | 95 (63 to 130) | 95.36 (62.15 to 136.95) |
| Miyazaki | 410 (337 to 496) | 923 (773 to 1105) | 125 (104.29 to 148.45) | 43 (29 to 62) | 88 (59 to 122) | 103.66 (71.6 to 143.34) |
| Kagoshima | 729 (604 to 880) | 1423 (1183 to 1696) | 95.21 (77.84 to 116.9) | 77 (49 to 111) | 136 (91 to 191) | 76.68 (49.09 to 112.76) |
| Okinawa | 315 (261 to 381) | 907 (757 to 1090) | 187.7 (167.53 to 208.96) | 33 (21 to 47) | 89 (58 to 125) | 171.45 (132.21 to 217.09) |
| DALYs, disability-adjusted life years; UI, uncertainty interval. | | | | | | |

| Table S2. Cases and rates (per 100,000 population) of vision impairment due to age-related macular degeneration prevalence, 1990–2021 | | | | | | |
| --- | --- | --- | --- | --- | --- | --- |
|  | Prevalence | | | | | |
|  | Overall (95% UI) | | Male (95% UI) | | Female (95% UI) | |
|  | Cases | Age-standardized rate (per 100,000) | Cases | Age-standardized rate (per 100,000) | Cases | Age-standardized rate (per 100,000) |
| 1990 | 36027 (29884 to 43516) | 22.30 (18.62 to 26.85) | 14127 (11580 to 17266) | 21.84 (18.28 to 26.55) | 21899 (18256 to 26150) | 22.56 (18.85 to 26.97) |
| 1991 | 37049 (30715 to 44690) | 22.01 (18.38 to 26.48) | 14528 (11931 to 17754) | 21.60 (18.05 to 26.25) | 22522 (18752 to 26848) | 22.23 (18.55 to 26.53) |
| 1992 | 38160 (31641 to 45975) | 21.74 (18.15 to 26.17) | 14957 (12313 to 18264) | 21.39 (17.85 to 25.98) | 23204 (19328 to 27683) | 21.93 (18.30 to 26.15) |
| 1993 | 39273 (32602 to 47298) | 21.50 (17.94 to 25.90) | 15380 (12651 to 18762) | 21.19 (17.67 to 25.78) | 23893 (19925 to 28522) | 21.66 (18.09 to 25.83) |
| 1994 | 40416 (33598 to 48667) | 21.30 (17.76 to 25.66) | 15813 (12995 to 19262) | 21.02 (17.51 to 25.60) | 24603 (20524 to 29388) | 21.44 (17.91 to 25.56) |
| 1995 | 41763 (34732 to 50258) | 21.13 (17.61 to 25.47) | 16308 (13392 to 19853) | 20.88 (17.37 to 25.46) | 25454 (21242 to 30413) | 21.26 (17.76 to 25.35) |
| 1996 | 43211 (35970 to 51935) | 21.00 (17.51 to 25.30) | 16841 (13841 to 20489) | 20.77 (17.32 to 25.30) | 26370 (22062 to 31532) | 21.12 (17.64 to 25.19) |
| 1997 | 44884 (37423 to 53944) | 20.91 (17.44 to 25.16) | 17471 (14376 to 21224) | 20.69 (17.26 to 25.18) | 27413 (22925 to 32764) | 21.00 (17.52 to 25.02) |
| 1998 | 46573 (38896 to 56066) | 20.82 (17.40 to 25.08) | 18106 (14904 to 21925) | 20.63 (17.21 to 25.08) | 28467 (23793 to 34008) | 20.90 (17.45 to 24.91) |
| 1999 | 48227 (40260 to 58142) | 20.74 (17.35 to 25.00) | 18726 (15436 to 22627) | 20.56 (17.13 to 24.95) | 29501 (24666 to 35260) | 20.81 (17.38 to 24.82) |
| 2000 | 50019 (41836 to 60428) | 20.66 (17.28 to 24.92) | 19379 (16014 to 23429) | 20.47 (17.05 to 24.82) | 30640 (25613 to 36649) | 20.73 (17.29 to 24.74) |
| 2001 | 51832 (43388 to 62643) | 20.56 (17.20 to 24.80) | 20014 (16592 to 24187) | 20.33 (16.96 to 24.64) | 31818 (26612 to 38094) | 20.66 (17.24 to 24.64) |
| 2002 | 53793 (45075 to 65002) | 20.45 (17.11 to 24.67) | 20684 (17202 to 24999) | 20.16 (16.85 to 24.39) | 33109 (27734 to 39669) | 20.60 (17.20 to 24.55) |
| 2003 | 55698 (46705 to 67284) | 20.34 (17.02 to 24.54) | 21319 (17782 to 25788) | 19.98 (16.70 to 24.15) | 34379 (28827 to 41220) | 20.54 (17.17 to 24.46) |
| 2004 | 57536 (48271 to 69473) | 20.23 (16.94 to 24.41) | 21949 (18356 to 26596) | 19.81 (16.57 to 23.95) | 35587 (29796 to 42749) | 20.48 (17.11 to 24.37) |
| 2005 | 59472 (49949 to 71870) | 20.14 (16.86 to 24.28) | 22646 (18869 to 27488) | 19.70 (16.53 to 23.80) | 36826 (30848 to 44293) | 20.41 (17.05 to 24.28) |
| 2006 | 61341 (51567 to 74185) | 20.04 (16.78 to 24.16) | 23376 (19497 to 28413) | 19.62 (16.43 to 23.72) | 37964 (31859 to 45599) | 20.30 (16.98 to 24.13) |
| 2007 | 63265 (53272 to 76532) | 19.92 (16.69 to 24.01) | 24188 (20221 to 29389) | 19.55 (16.39 to 23.64) | 39077 (32834 to 46870) | 20.15 (16.87 to 23.95) |
| 2008 | 65096 (54844 to 78709) | 19.81 (16.62 to 23.87) | 24985 (20887 to 30345) | 19.50 (16.36 to 23.58) | 40110 (33668 to 48020) | 20.00 (16.75 to 23.76) |
| 2009 | 66882 (56292 to 80854) | 19.71 (16.55 to 23.75) | 25774 (21497 to 31281) | 19.46 (16.36 to 23.53) | 41108 (34467 to 49115) | 19.87 (16.65 to 23.61) |
| 2010 | 68855 (57892 to 83191) | 19.67 (16.51 to 23.69) | 26594 (22215 to 32255) | 19.45 (16.35 to 23.52) | 42261 (35398 to 50458) | 19.80 (16.59 to 23.52) |
| 2011 | 70970 (59591 to 85543) | 19.68 (16.51 to 23.70) | 27408 (22860 to 33240) | 19.45 (16.33 to 23.50) | 43563 (36434 to 51996) | 19.83 (16.65 to 23.58) |
| 2012 | 73433 (61569 to 88313) | 19.75 (16.56 to 23.78) | 28310 (23603 to 34320) | 19.45 (16.31 to 23.50) | 45123 (37726 to 53976) | 19.94 (16.76 to 23.74) |
| 2013 | 75954 (63600 to 91403) | 19.84 (16.61 to 23.84) | 29200 (24283 to 35386) | 19.46 (16.30 to 23.49) | 46755 (39097 to 56013) | 20.08 (16.87 to 23.95) |
| 2014 | 78368 (65584 to 94379) | 19.92 (16.67 to 23.90) | 30066 (24991 to 36429) | 19.47 (16.27 to 23.49) | 48302 (40411 to 57891) | 20.20 (16.95 to 24.11) |
| 2015 | 80727 (67517 to 96976) | 19.96 (16.71 to 23.95) | 30941 (25712 to 37572) | 19.48 (16.24 to 23.48) | 49786 (41653 to 59634) | 20.26 (16.96 to 24.18) |
| 2016 | 82840 (69341 to 99444) | 19.93 (16.70 to 23.94) | 31805 (26530 to 38632) | 19.47 (16.25 to 23.52) | 51035 (42637 to 61190) | 20.22 (16.93 to 24.19) |
| 2017 | 85105 (71287 to 102115) | 19.86 (16.64 to 23.91) | 32800 (27354 to 39760) | 19.45 (16.25 to 23.50) | 52305 (43679 to 62656) | 20.13 (16.87 to 24.11) |
| 2018 | 87221 (73118 to 104624) | 19.79 (16.58 to 23.87) | 33733 (28121 to 40749) | 19.42 (16.23 to 23.43) | 53488 (44741 to 63894) | 20.03 (16.79 to 24.02) |
| 2019 | 89234 (74869 to 106865) | 19.74 (16.56 to 23.85) | 34574 (28839 to 41752) | 19.37 (16.22 to 23.42) | 54660 (45802 to 65231) | 19.98 (16.76 to 23.98) |
| 2020 | 91222 (76310 to 109617) | 19.70 (16.54 to 23.84) | 35139 (29236 to 42437) | 19.20 (16.08 to 23.32) | 56083 (47017 to 66910) | 20.04 (16.77 to 24.04) |
| 2021 | 93310 (78103 to 112033) | 19.73 (16.56 to 23.90) | 36375 (30344 to 44085) | 19.44 (16.31 to 23.59) | 56935 (47768 to 68506) | 19.89 (16.58 to 23.83) |
| UI, uncertainty interval. | | | | | | |

| Table S3. Cases and rates (per 100,000 population) of vision impairment due to age-related macular degeneration DALY, 1990–2021 | | | | | | |
| --- | --- | --- | --- | --- | --- | --- |
|  | Prevalence | | | | | |
|  | Overall (95% UI) | | Male (95% UI) | | Female (95% UI) | |
|  | Cases | Age-standardized rate (per 100,000) | Cases | Age-standardized rate (per 100,000) | Cases | Age-standardized rate (per 100,000) |
| 1990 | 3814 (2442 to 5349) | 2.34 (1.51 to 3.28) | 1549 (991 to 2181) | 2.34 (1.51 to 3.29) | 2265 (1469 to 3200) | 2.33 (1.51 to 3.30) |
| 1991 | 3905 (2517 to 5517) | 2.30 (1.49 to 3.24) | 1588 (1010 to 2255) | 2.31 (1.49 to 3.25) | 2317 (1503 to 3280) | 2.29 (1.49 to 3.24) |
| 1992 | 3996 (2581 to 5641) | 2.26 (1.47 to 3.17) | 1627 (1034 to 2280) | 2.28 (1.48 to 3.18) | 2369 (1552 to 3333) | 2.25 (1.47 to 3.16) |
| 1993 | 4093 (2648 to 5750) | 2.23 (1.45 to 3.11) | 1666 (1061 to 2340) | 2.25 (1.45 to 3.14) | 2427 (1568 to 3425) | 2.21 (1.44 to 3.12) |
| 1994 | 4185 (2712 to 5881) | 2.20 (1.42 to 3.08) | 1705 (1089 to 2390) | 2.22 (1.43 to 3.09) | 2479 (1616 to 3457) | 2.17 (1.41 to 3.05) |
| 1995 | 4295 (2780 to 5985) | 2.17 (1.41 to 3.02) | 1746 (1095 to 2444) | 2.20 (1.40 to 3.05) | 2549 (1673 to 3575) | 2.15 (1.40 to 3.01) |
| 1996 | 4421 (2844 to 6164) | 2.15 (1.39 to 3.01) | 1794 (1138 to 2536) | 2.18 (1.42 to 3.04) | 2627 (1691 to 3653) | 2.12 (1.37 to 2.98) |
| 1997 | 4565 (2965 to 6368) | 2.13 (1.39 to 2.97) | 1850 (1186 to 2603) | 2.16 (1.40 to 3.04) | 2715 (1791 to 3794) | 2.10 (1.38 to 2.93) |
| 1998 | 4702 (3083 to 6510) | 2.11 (1.38 to 2.92) | 1902 (1202 to 2668) | 2.14 (1.38 to 2.97) | 2799 (1843 to 3892) | 2.08 (1.37 to 2.91) |
| 1999 | 4844 (3169 to 6765) | 2.09 (1.37 to 2.92) | 1958 (1234 to 2743) | 2.12 (1.37 to 2.95) | 2886 (1901 to 4036) | 2.06 (1.35 to 2.92) |
| 2000 | 4995 (3260 to 6863) | 2.07 (1.35 to 2.86) | 2012 (1275 to 2795) | 2.10 (1.35 to 2.91) | 2983 (1971 to 4145) | 2.05 (1.34 to 2.86) |
| 2001 | 5151 (3365 to 7105) | 2.06 (1.34 to 2.84) | 2071 (1329 to 2884) | 2.09 (1.35 to 2.88) | 3080 (2023 to 4252) | 2.03 (1.33 to 2.83) |
| 2002 | 5323 (3512 to 7350) | 2.04 (1.34 to 2.83) | 2133 (1358 to 2956) | 2.06 (1.33 to 2.85) | 3190 (2113 to 4424) | 2.02 (1.33 to 2.83) |
| 2003 | 5491 (3629 to 7569) | 2.03 (1.33 to 2.81) | 2194 (1412 to 3043) | 2.04 (1.33 to 2.84) | 3297 (2193 to 4563) | 2.01 (1.32 to 2.80) |
| 2004 | 5651 (3751 to 7797) | 2.01 (1.31 to 2.77) | 2253 (1450 to 3128) | 2.02 (1.31 to 2.81) | 3398 (2258 to 4655) | 2.00 (1.32 to 2.78) |
| 2005 | 5822 (3851 to 8108) | 2.00 (1.31 to 2.80) | 2315 (1504 to 3232) | 2.01 (1.31 to 2.79) | 3507 (2328 to 4856) | 1.99 (1.30 to 2.79) |
| 2006 | 5979 (3976 to 8274) | 1.98 (1.30 to 2.75) | 2380 (1553 to 3331) | 2.00 (1.30 to 2.79) | 3599 (2392 to 4924) | 1.97 (1.30 to 2.75) |
| 2007 | 6146 (4057 to 8440) | 1.97 (1.27 to 2.73) | 2458 (1587 to 3407) | 1.99 (1.28 to 2.76) | 3688 (2453 to 5063) | 1.95 (1.28 to 2.72) |
| 2008 | 6301 (4186 to 8688) | 1.95 (1.27 to 2.71) | 2533 (1625 to 3537) | 1.98 (1.28 to 2.77) | 3768 (2521 to 5155) | 1.93 (1.27 to 2.68) |
| 2009 | 6456 (4295 to 8948) | 1.94 (1.27 to 2.69) | 2604 (1673 to 3634) | 1.98 (1.27 to 2.73) | 3853 (2566 to 5296) | 1.91 (1.25 to 2.66) |
| 2010 | 6635 (4421 to 9164) | 1.94 (1.27 to 2.69) | 2683 (1733 to 3692) | 1.97 (1.27 to 2.72) | 3952 (2641 to 5443) | 1.90 (1.25 to 2.66) |
| 2011 | 6820 (4535 to 9448) | 1.94 (1.27 to 2.69) | 2755 (1793 to 3848) | 1.97 (1.29 to 2.74) | 4065 (2723 to 5581) | 1.90 (1.26 to 2.67) |
| 2012 | 7032 (4654 to 9728) | 1.94 (1.27 to 2.69) | 2839 (1856 to 3968) | 1.97 (1.29 to 2.74) | 4194 (2796 to 5744) | 1.91 (1.24 to 2.66) |
| 2013 | 7242 (4865 to 9946) | 1.94 (1.27 to 2.67) | 2917 (1885 to 4045) | 1.97 (1.26 to 2.73) | 4325 (2935 to 5946) | 1.91 (1.27 to 2.64) |
| 2014 | 7451 (4969 to 10308) | 1.94 (1.28 to 2.70) | 2994 (1934 to 4196) | 1.97 (1.26 to 2.75) | 4457 (3002 to 6110) | 1.92 (1.27 to 2.67) |
| 2015 | 7667 (5142 to 10606) | 1.95 (1.27 to 2.70) | 3079 (2009 to 4272) | 1.97 (1.28 to 2.73) | 4588 (3098 to 6286) | 1.92 (1.26 to 2.67) |
| 2016 | 7874 (5286 to 10934) | 1.95 (1.28 to 2.71) | 3160 (2053 to 4411) | 1.97 (1.27 to 2.76) | 4714 (3175 to 6470) | 1.92 (1.28 to 2.68) |
| 2017 | 8112 (5439 to 11185) | 1.95 (1.28 to 2.68) | 3264 (2152 to 4515) | 1.97 (1.28 to 2.73) | 4848 (3268 to 6654) | 1.91 (1.27 to 2.63) |
| 2018 | 8336 (5606 to 11515) | 1.94 (1.27 to 2.71) | 3355 (2204 to 4685) | 1.97 (1.28 to 2.74) | 4981 (3381 to 6848) | 1.91 (1.26 to 2.66) |
| 2019 | 8549 (5791 to 11755) | 1.94 (1.29 to 2.69) | 3441 (2276 to 4787) | 1.97 (1.29 to 2.74) | 5108 (3470 to 7053) | 1.91 (1.27 to 2.62) |
| 2020 | 8771 (5860 to 12081) | 1.95 (1.28 to 2.68) | 3502 (2300 to 4897) | 1.96 (1.27 to 2.73) | 5269 (3535 to 7303) | 1.93 (1.27 to 2.68) |
| 2021 | 8907 (5984 to 12298) | 1.94 (1.27 to 2.66) | 3597 (2407 to 5005) | 1.97 (1.29 to 2.72) | 5309 (3575 to 7403) | 1.91 (1.26 to 2.63) |
| DALYs, disability-adjusted life years. UI, uncertainty interval. | | | | | | |

| Table S4. Age-standardized percentage and percentage change of prevalence of vision impairment due to age-related macular degeneration by age and sex, 1990–2021 | | | |
| --- | --- | --- | --- |
|  | Prevalence | | |
|  | Overall (95% UI) | Male (95% UI) | Female (95% UI) |
|  | Rate (per 100,000) | Rate (per 100,000) | Rate (per 100,000) |
| **Japan** | -11.52 (-14.1 to -9.13) | -10.97 (-13.39 to -8.34) | -11.82 (-14.65 to -9.06) |
| **Age** |  |  |  |
| 45-49 years | -17.35 (-28.02 to -4.12) | -17.45 (-27.75 to -3.68) | -17.58 (-31.37 to -1.69) |
| 50-54 years | -18.18 (-25.03 to -9.35) | -17.34 (-24.81 to -7.65) | -19.36 (-27.65 to -9.44) |
| 55-59 years | -16.83 (-21.88 to -10.83) | -15.53 (-21.01 to -9.48) | -18.29 (-24.35 to -11.58) |
| 60-64 years | -14.53 (-19 to -9.28) | -13.16 (-17.74 to -7.7) | -15.87 (-20.78 to -9.96) |
| 65-69 years | -12.7 (-16.56 to -8.55) | -11 (-14.91 to -6.99) | -13.78 (-18.36 to -9.09) |
| 70-74 years | -11.72 (-15.32 to -8.27) | -10.07 (-13.81 to -6.2) | -12.43 (-16.62 to -8.31) |
| 75-79 years | -11.11 (-14.65 to -8.33) | -9.9 (-13.36 to -6.49) | -11.49 (-15.31 to -8.1) |
| 80-84 years | -10.52 (-13.55 to -7.86) | -10.02 (-13.16 to -6.91) | -10.45 (-13.79 to -7.43) |
| 85-89 years | -9.62 (-12.18 to -6.96) | -10.37 (-13.19 to -7.48) | -9.07 (-11.96 to -6.06) |
| 90-94 years | -8.44 (-10.63 to -6) | -10.22 (-12.94 to -7.71) | -7.66 (-10.28 to -4.99) |
| 95+ years | -6.96 (-9.06 to -4.7) | -9.1 (-11.89 to -6.56) | -6.24 (-8.46 to -3.95) |
| UI, uncertainty interval. | | | |

| Table S5. Age-standardized percentage and percentage change of DALYs of vision impairment due to age-related macular degeneration by age and sex, 1990–2021 | | | |
| --- | --- | --- | --- |
|  | DALYs | | |
|  | Overall (95% UI) | Male (95% UI) | Female (95% UI) |
|  | Rate (per 100,000) | Rate (per 100,000) | Rate (per 100,000) |
| **Japan** | -17.25 (-21.25 to -13.38) | -16.03 (-20.75 to -11.26) | -18.4 (-23.13 to -13.24) |
| **Age** |  |  |  |
| 45-49 years | -24.76 (-35.42 to -12.53) | -24.18 (-35.02 to -8.93) | -25.95 (-41.45 to -7.99) |
| 50-54 years | -25.54 (-32.03 to -18.02) | -24 (-31.1 to -14.66) | -27.64 (-35.45 to -18.7) |
| 55-59 years | -22.81 (-37.28 to -4.17) | -21.16 (-41.54 to 5.5) | -24.75 (-41.07 to -1.35) |
| 60-64 years | -20.76 (-31.41 to -8.58) | -18.58 (-34.23 to -0.37) | -23.09 (-37.44 to -6.08) |
| 65-69 years | -19.14 (-29.09 to -9.01) | -16.15 (-31.38 to 1.79) | -21.61 (-33.66 to -8.41) |
| 70-74 years | -17.85 (-26.2 to -7.72) | -15.04 (-27.21 to -0.72) | -20.06 (-30.48 to -6.52) |
| 75-79 years | -16.73 (-25.02 to -7.12) | -14.81 (-25.15 to -2.49) | -18.09 (-29.48 to -6.59) |
| 80-84 years | -15.94 (-21.83 to -9.36) | -14.92 (-23.63 to -5.13) | -16.5 (-24.48 to -8.26) |
| 85-89 years | -14.16 (-18.92 to -9.08) | -14.65 (-21.17 to -6.44) | -13.87 (-20.3 to -7.39) |
| 90-94 years | -11.88 (-16.51 to -6.97) | -13.85 (-19.51 to -7.16) | -11.05 (-16.95 to -4.04) |
| 95+ years | -9.43 (-13.1 to -5.19) | -12.4 (-17.43 to -6.97) | -8.41 (-13.12 to -3.59) |
| DALYs, disability-adjusted life years. UI, uncertainty interval. | | | |

| Table S6. Cases and age-standardized rates (per 100,000 population) of prevalence in vision impairment due to age-macular degeneration in, 1990-2021 | | | | | | | | |
| --- | --- | --- | --- | --- | --- | --- | --- | --- |
| Prevalence of vision impairment | | | | | | | | |
|  | Blindness and vision impairment | | Moderate vision impairment | | Severe vision impairment | | Blindness | |
|  | Cases (95% UI) | Rates (95% UI) | Cases (95% UI) | Rates (95% UI) | Cases (95% UI) | Rates (95% UI) | Cases (95% UI) | Rates (95% UI) |
| 1990 | 36027 (29884 to 43516) | 22.30 (18.62 to 26.85) | 15288 (11537 to 19753) | 9.49 (7.20 to 12.19) | 3617 (2635 to 4756) | 2.18 (1.62 to 2.86) | 17122 (12588 to 22831) | 10.63 (7.98 to 14.32) |
| 1991 | 37049 (30715 to 44690) | 22.01 (18.38 to 26.48) | 15892 (11983 to 20549) | 9.46 (7.19 to 12.16) | 3731 (2719 to 4898) | 2.16 (1.60 to 2.84) | 17426 (12817 to 23232) | 10.38 (7.77 to 14.00) |
| 1992 | 38160 (31641 to 45975) | 21.74 (18.15 to 26.17) | 16546 (12488 to 21407) | 9.45 (7.18 to 12.14) | 3854 (2811 to 5050) | 2.15 (1.59 to 2.81) | 17760 (13070 to 23702) | 10.14 (7.58 to 13.69) |
| 1993 | 39273 (32602 to 47298) | 21.50 (17.94 to 25.90) | 17207 (13000 to 22300) | 9.43 (7.18 to 12.11) | 3981 (2904 to 5214) | 2.14 (1.58 to 2.80) | 18085 (13318 to 24166) | 9.93 (7.40 to 13.41) |
| 1994 | 40416 (33598 to 48667) | 21.30 (17.76 to 25.66) | 17883 (13523 to 23155) | 9.43 (7.16 to 12.10) | 4115 (3007 to 5393) | 2.13 (1.58 to 2.79) | 18418 (13572 to 24657) | 9.74 (7.23 to 13.15) |
| 1995 | 41763 (34732 to 50258) | 21.13 (17.61 to 25.47) | 18650 (14105 to 24139) | 9.43 (7.14 to 12.11) | 4272 (3127 to 5608) | 2.13 (1.58 to 2.78) | 18841 (13884 to 25283) | 9.57 (7.10 to 12.93) |
| 1996 | 43211 (35970 to 51935) | 21.00 (17.51 to 25.30) | 19468 (14748 to 25199) | 9.44 (7.17 to 12.12) | 4447 (3261 to 5835) | 2.13 (1.58 to 2.79) | 19296 (14266 to 25966) | 9.43 (6.99 to 12.75) |
| 1997 | 44884 (37423 to 53944) | 20.91 (17.44 to 25.16) | 20400 (15483 to 26427) | 9.48 (7.21 to 12.18) | 4649 (3414 to 6090) | 2.14 (1.58 to 2.80) | 19835 (14700 to 26788) | 9.29 (6.89 to 12.58) |
| 1998 | 46573 (38896 to 56066) | 20.82 (17.40 to 25.08) | 21349 (16196 to 27687) | 9.51 (7.25 to 12.24) | 4855 (3569 to 6345) | 2.15 (1.59 to 2.82) | 20368 (15116 to 27610) | 9.16 (6.79 to 12.41) |
| 1999 | 48227 (40260 to 58142) | 20.74 (17.35 to 25.00) | 22280 (16867 to 28927) | 9.54 (7.27 to 12.30) | 5054 (3719 to 6611) | 2.16 (1.60 to 2.83) | 20893 (15510 to 28415) | 9.04 (6.69 to 12.25) |
| 2000 | 50019 (41836 to 60428) | 20.66 (17.28 to 24.92) | 23271 (17624 to 30233) | 9.56 (7.28 to 12.34) | 5257 (3876 to 6892) | 2.17 (1.60 to 2.83) | 21491 (15962 to 29288) | 8.93 (6.61 to 12.12) |
| 2001 | 51832 (43388 to 62643) | 20.56 (17.20 to 24.80) | 24264 (18389 to 31458) | 9.56 (7.28 to 12.32) | 5451 (4031 to 7160) | 2.17 (1.60 to 2.83) | 22118 (16414 to 30299) | 8.83 (6.53 to 11.98) |
| 2002 | 53793 (45075 to 65002) | 20.45 (17.11 to 24.67) | 25312 (19177 to 32736) | 9.55 (7.27 to 12.30) | 5648 (4188 to 7428) | 2.16 (1.59 to 2.82) | 22832 (16926 to 31298) | 8.74 (6.46 to 11.84) |
| 2003 | 55698 (46705 to 67284) | 20.34 (17.02 to 24.54) | 26334 (19911 to 33969) | 9.53 (7.26 to 12.27) | 5835 (4329 to 7682) | 2.15 (1.58 to 2.81) | 23529 (17447 to 32178) | 8.66 (6.40 to 11.71) |
| 2004 | 57536 (48271 to 69473) | 20.23 (16.94 to 24.41) | 27322 (20660 to 35146) | 9.52 (7.24 to 12.24) | 6015 (4454 to 7911) | 2.14 (1.58 to 2.80) | 24199 (17980 to 32965) | 8.57 (6.34 to 11.59) |
| 2005 | 59472 (49949 to 71870) | 20.14 (16.86 to 24.28) | 28372 (21448 to 36417) | 9.51 (7.23 to 12.23) | 6210 (4593 to 8160) | 2.13 (1.58 to 2.79) | 24891 (18530 to 33762) | 8.50 (6.29 to 11.47) |
| 2006 | 61341 (51567 to 74185) | 20.04 (16.78 to 24.16) | 29395 (22254 to 37704) | 9.49 (7.22 to 12.19) | 6418 (4747 to 8431) | 2.14 (1.58 to 2.79) | 25527 (18974 to 34519) | 8.41 (6.22 to 11.36) |
| 2007 | 63265 (53272 to 76532) | 19.92 (16.69 to 24.01) | 30440 (23051 to 39023) | 9.46 (7.20 to 12.15) | 6655 (4912 to 8729) | 2.14 (1.58 to 2.80) | 26170 (19431 to 35432) | 8.31 (6.15 to 11.23) |
| 2008 | 65096 (54844 to 78709) | 19.81 (16.62 to 23.87) | 31432 (23737 to 40377) | 9.43 (7.18 to 12.11) | 6886 (5074 to 9033) | 2.15 (1.58 to 2.81) | 26778 (19831 to 36299) | 8.22 (6.08 to 11.11) |
| 2009 | 66882 (56292 to 80854) | 19.71 (16.55 to 23.75) | 32387 (24388 to 41697) | 9.41 (7.16 to 12.09) | 7104 (5236 to 9345) | 2.15 (1.58 to 2.81) | 27391 (20253 to 37175) | 8.15 (6.02 to 11.02) |
| 2010 | 68855 (57892 to 83191) | 19.67 (16.51 to 23.69) | 33414 (25084 to 43121) | 9.40 (7.15 to 12.10) | 7308 (5394 to 9621) | 2.15 (1.59 to 2.82) | 28133 (20791 to 38131) | 8.11 (5.98 to 10.97) |
| 2011 | 70970 (59591 to 85543) | 19.68 (16.51 to 23.70) | 34547 (25851 to 44519) | 9.43 (7.18 to 12.14) | 7483 (5524 to 9842) | 2.15 (1.58 to 2.81) | 28940 (21421 to 39259) | 8.10 (5.97 to 10.96) |
| 2012 | 73433 (61569 to 88313) | 19.75 (16.56 to 23.78) | 35913 (26816 to 46262) | 9.51 (7.24 to 12.23) | 7664 (5661 to 10070) | 2.14 (1.58 to 2.80) | 29856 (22164 to 40374) | 8.11 (5.96 to 10.98) |
| 2013 | 75954 (63600 to 91403) | 19.84 (16.61 to 23.84) | 37318 (27880 to 48160) | 9.59 (7.32 to 12.34) | 7830 (5790 to 10278) | 2.13 (1.57 to 2.79) | 30807 (22952 to 41554) | 8.12 (5.97 to 11.02) |
| 2014 | 78368 (65584 to 94379) | 19.92 (16.67 to 23.90) | 38629 (28891 to 50030) | 9.66 (7.38 to 12.42) | 7988 (5916 to 10513) | 2.12 (1.57 to 2.78) | 31751 (23604 to 42710) | 8.14 (5.98 to 11.03) |
| 2015 | 80727 (67517 to 96976) | 19.96 (16.71 to 23.95) | 39805 (29778 to 51728) | 9.69 (7.41 to 12.48) | 8155 (6045 to 10744) | 2.12 (1.56 to 2.78) | 32766 (24313 to 43949) | 8.15 (5.98 to 11.06) |
| 2016 | 82840 (69341 to 99444) | 19.93 (16.70 to 23.94) | 40735 (30442 to 52985) | 9.66 (7.38 to 12.45) | 8310 (6166 to 10879) | 2.11 (1.56 to 2.77) | 33795 (25052 to 45102) | 8.16 (6.00 to 11.08) |
| 2017 | 85105 (71287 to 102115) | 19.86 (16.64 to 23.91) | 41644 (31081 to 54206) | 9.60 (7.33 to 12.36) | 8475 (6292 to 11054) | 2.11 (1.56 to 2.76) | 34987 (25892 to 46539) | 8.16 (6.02 to 11.09) |
| 2018 | 87221 (73118 to 104624) | 19.79 (16.58 to 23.87) | 42425 (31621 to 55229) | 9.52 (7.27 to 12.28) | 8628 (6408 to 11256) | 2.10 (1.55 to 2.75) | 36168 (26763 to 48248) | 8.16 (6.03 to 11.11) |
| 2019 | 89234 (74869 to 106865) | 19.74 (16.56 to 23.85) | 43176 (32124 to 56247) | 9.47 (7.21 to 12.21) | 8785 (6525 to 11477) | 2.10 (1.55 to 2.75) | 37273 (27628 to 49642) | 8.18 (6.04 to 11.12) |
| 2020 | 91222 (76310 to 109617) | 19.70 (16.54 to 23.84) | 43798 (32468 to 57262) | 9.38 (7.14 to 12.06) | 8973 (6663 to 11775) | 2.10 (1.56 to 2.76) | 38451 (28583 to 51201) | 8.22 (6.09 to 11.19) |
| 2021 | 93310 (78103 to 112033) | 19.73 (16.56 to 23.90) | 45186 (33668 to 58849) | 9.47 (7.13 to 12.20) | 9186 (6801 to 12056) | 2.11 (1.56 to 2.76) | 38938 (28979 to 51898) | 8.14 (6.02 to 11.09) |
| UI, uncertainty interval. | | | | | | | | |

| Table S7. Cases and age-standardized rates (per 100,000 population) of DALYs in vision impairment due to age-macular degeneration, 1990-2021 | | | | | | | | |
| --- | --- | --- | --- | --- | --- | --- | --- | --- |
| DALYs of vision impairment | | | | | | | | |
|  | Blindness and vision impairment | | Moderate vision impairment | | Severe vision impairment | | Blindness | |
|  | Cases (95% UI) | Rates (95% UI) | Cases (95% UI) | Rates (95% UI) | Cases (95% UI) | Rates (95% UI) | Cases (95% UI) | Rates (95% UI) |
| 1990 | 4223 (2640 to 6067) | 2.60 (1.64 to 3.71) | 463 (261 to 775) | 0.29 (0.16 to 0.48) | 647 (410 to 1002) | 0.39 (0.25 to 0.60) | 3114 (1764 to 4683) | 1.93 (1.10 to 2.84) |
| 1991 | 4319 (2736 to 6198) | 2.56 (1.62 to 3.64) | 481 (269 to 800) | 0.29 (0.16 to 0.47) | 667 (418 to 1033) | 0.39 (0.24 to 0.59) | 3171 (1806 to 4748) | 1.88 (1.08 to 2.77) |
| 1992 | 4419 (2787 to 6317) | 2.51 (1.59 to 3.57) | 501 (282 to 839) | 0.29 (0.16 to 0.47) | 689 (430 to 1057) | 0.38 (0.24 to 0.59) | 3229 (1828 to 4868) | 1.84 (1.05 to 2.74) |
| 1993 | 4522 (2859 to 6488) | 2.47 (1.57 to 3.54) | 520 (293 to 876) | 0.29 (0.16 to 0.48) | 711 (445 to 1114) | 0.38 (0.24 to 0.59) | 3291 (1848 to 4989) | 1.80 (1.02 to 2.69) |
| 1994 | 4630 (2895 to 6622) | 2.44 (1.53 to 3.47) | 541 (304 to 921) | 0.28 (0.16 to 0.48) | 736 (460 to 1134) | 0.38 (0.24 to 0.59) | 3353 (1877 to 5054) | 1.77 (1.00 to 2.65) |
| 1995 | 4755 (2988 to 6813) | 2.40 (1.51 to 3.43) | 564 (318 to 959) | 0.29 (0.16 to 0.48) | 763 (477 to 1178) | 0.38 (0.24 to 0.59) | 3428 (1922 to 5153) | 1.74 (0.98 to 2.61) |
| 1996 | 4891 (3088 to 6987) | 2.38 (1.51 to 3.40) | 589 (334 to 991) | 0.29 (0.16 to 0.48) | 794 (491 to 1234) | 0.38 (0.24 to 0.59) | 3508 (1956 to 5211) | 1.71 (0.96 to 2.54) |
| 1997 | 5053 (3172 to 7188) | 2.36 (1.48 to 3.36) | 617 (348 to 1034) | 0.29 (0.16 to 0.48) | 829 (520 to 1270) | 0.38 (0.24 to 0.59) | 3607 (2016 to 5367) | 1.69 (0.95 to 2.51) |
| 1998 | 5220 (3315 to 7408) | 2.34 (1.49 to 3.33) | 647 (368 to 1070) | 0.29 (0.16 to 0.48) | 867 (547 to 1329) | 0.38 (0.24 to 0.59) | 3706 (2089 to 5450) | 1.67 (0.94 to 2.46) |
| 1999 | 5374 (3410 to 7636) | 2.32 (1.47 to 3.31) | 675 (380 to 1110) | 0.29 (0.16 to 0.47) | 903 (560 to 1396) | 0.39 (0.24 to 0.60) | 3797 (2139 to 5651) | 1.64 (0.92 to 2.44) |
| 2000 | 5548 (3565 to 7863) | 2.30 (1.47 to 3.28) | 704 (402 to 1165) | 0.29 (0.16 to 0.48) | 940 (583 to 1465) | 0.39 (0.24 to 0.60) | 3905 (2224 to 5781) | 1.62 (0.91 to 2.41) |
| 2001 | 5723 (3675 to 8111) | 2.28 (1.47 to 3.24) | 734 (415 to 1217) | 0.29 (0.16 to 0.48) | 972 (606 to 1513) | 0.39 (0.24 to 0.60) | 4017 (2310 to 5929) | 1.61 (0.92 to 2.39) |
| 2002 | 5919 (3766 to 8312) | 2.26 (1.45 to 3.20) | 766 (436 to 1260) | 0.29 (0.16 to 0.48) | 1007 (635 to 1561) | 0.39 (0.25 to 0.60) | 4147 (2386 to 6103) | 1.59 (0.90 to 2.36) |
| 2003 | 6111 (3914 to 8620) | 2.25 (1.44 to 3.18) | 796 (452 to 1311) | 0.29 (0.16 to 0.48) | 1041 (650 to 1587) | 0.38 (0.24 to 0.58) | 4274 (2490 to 6291) | 1.57 (0.91 to 2.36) |
| 2004 | 6285 (4042 to 8826) | 2.23 (1.42 to 3.14) | 825 (469 to 1375) | 0.29 (0.16 to 0.48) | 1071 (670 to 1655) | 0.38 (0.24 to 0.59) | 4389 (2569 to 6435) | 1.56 (0.90 to 2.31) |
| 2005 | 6476 (4155 to 9088) | 2.21 (1.42 to 3.12) | 858 (490 to 1428) | 0.29 (0.16 to 0.48) | 1107 (681 to 1695) | 0.38 (0.24 to 0.58) | 4512 (2651 to 6618) | 1.54 (0.89 to 2.29) |
| 2006 | 6659 (4294 to 9397) | 2.20 (1.40 to 3.09) | 889 (510 to 1475) | 0.29 (0.16 to 0.48) | 1143 (712 to 1750) | 0.38 (0.24 to 0.58) | 4628 (2722 to 6798) | 1.53 (0.89 to 2.27) |
| 2007 | 6839 (4443 to 9628) | 2.18 (1.40 to 3.07) | 920 (529 to 1543) | 0.29 (0.16 to 0.48) | 1184 (746 to 1822) | 0.38 (0.24 to 0.59) | 4734 (2777 to 6944) | 1.51 (0.87 to 2.24) |
| 2008 | 7017 (4565 to 9904) | 2.16 (1.38 to 3.06) | 949 (541 to 1586) | 0.29 (0.16 to 0.47) | 1224 (776 to 1889) | 0.38 (0.24 to 0.59) | 4844 (2831 to 7098) | 1.49 (0.86 to 2.23) |
| 2009 | 7206 (4698 to 10077) | 2.15 (1.38 to 3.05) | 979 (560 to 1638) | 0.28 (0.16 to 0.47) | 1264 (796 to 1928) | 0.38 (0.24 to 0.59) | 4963 (2871 to 7299) | 1.48 (0.85 to 2.22) |
| 2010 | 7405 (4821 to 10359) | 2.14 (1.37 to 3.04) | 1009 (575 to 1696) | 0.28 (0.16 to 0.47) | 1300 (817 to 2010) | 0.38 (0.24 to 0.59) | 5096 (3014 to 7446) | 1.47 (0.85 to 2.21) |
| 2011 | 7616 (4969 to 10676) | 2.14 (1.37 to 3.02) | 1043 (591 to 1749) | 0.29 (0.16 to 0.47) | 1332 (841 to 2068) | 0.38 (0.24 to 0.59) | 5241 (3085 to 7629) | 1.47 (0.85 to 2.18) |
| 2012 | 7849 (5115 to 11050) | 2.14 (1.37 to 3.05) | 1085 (620 to 1813) | 0.29 (0.16 to 0.48) | 1362 (866 to 2118) | 0.38 (0.24 to 0.59) | 5402 (3217 to 7913) | 1.47 (0.85 to 2.17) |
| 2013 | 8088 (5285 to 11441) | 2.15 (1.38 to 3.04) | 1126 (645 to 1902) | 0.29 (0.16 to 0.49) | 1393 (881 to 2161) | 0.38 (0.24 to 0.59) | 5570 (3310 to 8181) | 1.47 (0.85 to 2.20) |
| 2014 | 8327 (5468 to 11814) | 2.15 (1.38 to 3.07) | 1166 (668 to 1950) | 0.29 (0.16 to 0.49) | 1424 (891 to 2194) | 0.38 (0.24 to 0.58) | 5738 (3437 to 8369) | 1.48 (0.85 to 2.20) |
| 2015 | 8572 (5640 to 12136) | 2.15 (1.38 to 3.04) | 1201 (690 to 2007) | 0.29 (0.17 to 0.49) | 1452 (910 to 2213) | 0.38 (0.24 to 0.58) | 5919 (3536 to 8672) | 1.48 (0.85 to 2.22) |
| 2016 | 8813 (5802 to 12463) | 2.15 (1.38 to 3.07) | 1229 (704 to 2037) | 0.29 (0.17 to 0.48) | 1478 (932 to 2273) | 0.38 (0.24 to 0.59) | 6106 (3623 to 8932) | 1.48 (0.85 to 2.19) |
| 2017 | 9078 (5961 to 12891) | 2.15 (1.38 to 3.04) | 1257 (715 to 2085) | 0.29 (0.16 to 0.48) | 1508 (956 to 2363) | 0.38 (0.24 to 0.59) | 6312 (3751 to 9276) | 1.48 (0.85 to 2.20) |
| 2018 | 9335 (6103 to 13365) | 2.15 (1.38 to 3.05) | 1281 (731 to 2100) | 0.29 (0.16 to 0.48) | 1535 (962 to 2378) | 0.38 (0.23 to 0.59) | 6519 (3888 to 9596) | 1.48 (0.86 to 2.18) |
| 2019 | 9584 (6279 to 13741) | 2.15 (1.38 to 3.05) | 1303 (741 to 2144) | 0.29 (0.16 to 0.48) | 1563 (982 to 2428) | 0.38 (0.23 to 0.59) | 6718 (4002 to 9865) | 1.48 (0.86 to 2.22) |
| 2020 | 9839 (6394 to 14099) | 2.15 (1.38 to 3.06) | 1321 (753 to 2159) | 0.28 (0.16 to 0.47) | 1594 (1003 to 2449) | 0.38 (0.24 to 0.58) | 6924 (4076 to 10219) | 1.49 (0.84 to 2.22) |
| 2021 | 10003 (6540 to 14275) | 2.14 (1.38 to 3.03) | 1363 (771 to 2240) | 0.29 (0.16 to 0.48) | 1631 (1022 to 2541) | 0.38 (0.24 to 0.59) | 7009 (4198 to 10383) | 1.48 (0.84 to 2.18) |
| DALYs, disability-adjusted life years; UI, uncertainty interval. | | | | | | | | |

| Table S8. Cases and rates per 100,000 of the population and percentage change of prevalence for age-related macular degeneration by sex and age, 1990–2021 | | | | | |
| --- | --- | --- | --- | --- | --- |
|  | 1990 | | 2021 | | 1990 to 2021 |
|  | Cases (95% UI) | Rates (95% UI) | Cases (95% UI) | Rates (95% UI) | Percentage change, % (95%UI) |
| **Global** | 3640180 (3037098 to 4353902) | 99.5 (83.16 to 118.04) | 8057521 (6705284 to 9823238) | 94 (78.32 to 114.42) | -5.53 (-8.84 to -2.23) |
| **Japan** | 36027 (29884 to 43516) | 22.3 (18.62 to 26.85) | 93310 (78103 to 112033) | 19.73 (16.56 to 23.9) | -11.52 (-14.1 to -9.13) |
| **Sex** |  |  |  |  |  |
| Male | 14127 (11580 to 17266) | 21.84 (18.28 to 26.55) | 36375 (30344 to 44085) | 19.44 (16.31 to 23.59) | -10.97 (-13.39 to -8.34) |
| Female | 21899 (18256 to 26150) | 22.56 (18.85 to 26.97) | 56935 (47768 to 68506) | 19.89 (16.58 to 23.83) | -11.82 (-14.65 to -9.06) |
| **Age** |  |  |  |  |  |
| 45-49 years | 155 (74 to 283) | 1.68 (0.81 to 3.08) | 138 (69 to 248) | 1.39 (0.69 to 2.5) | -17.35 (-28.02 to -4.12) |
| 50-54 years | 891 (524 to 1363) | 10.85 (6.39 to 16.6) | 832 (490 to 1267) | 8.88 (5.23 to 13.52) | -18.18 (-25.03 to -9.35) |
| 55-59 years | 2173 (1471 to 3064) | 27.86 (18.86 to 39.29) | 1829 (1256 to 2515) | 23.17 (15.91 to 31.85) | -16.83 (-21.88 to -10.83) |
| 60-64 years | 3545 (2435 to 4866) | 52.18 (35.83 to 71.61) | 3326 (2340 to 4520) | 44.6 (31.38 to 60.61) | -14.53 (-19 to -9.28) |
| 65-69 years | 4465 (3347 to 5972) | 86.81 (65.07 to 116.11) | 6022 (4546 to 7959) | 75.78 (57.2 to 100.15) | -12.7 (-16.56 to -8.55) |
| 70-74 years | 5299 (4072 to 6842) | 137.74 (105.86 to 177.86) | 11865 (9118 to 15245) | 121.6 (93.45 to 156.24) | -11.72 (-15.32 to -8.27) |
| 75-79 years | 6723 (5202 to 8671) | 221.36 (171.27 to 285.49) | 13367 (10292 to 17174) | 196.78 (151.5 to 252.82) | -11.11 (-14.65 to -8.33) |
| 80-84 years | 6206 (4800 to 7971) | 336.49 (260.25 to 432.19) | 16879 (13064 to 21486) | 301.1 (233.04 to 383.28) | -10.52 (-13.55 to -7.86) |
| 85-89 years | 4173 (3275 to 5334) | 497.98 (390.78 to 636.48) | 17483 (13600 to 22155) | 450.08 (350.11 to 570.36) | -9.62 (-12.18 to -6.96) |
| 90-94 years | 1846 (1445 to 2391) | 713.93 (558.61 to 924.75) | 12939 (10000 to 16827) | 653.71 (505.23 to 850.16) | -8.44 (-10.63 to -6) |
| 95+ years | 550 (407 to 730) | 1051.47 (777.18 to 1394.17) | 8628 (6447 to 11382) | 978.28 (731.05 to 1290.56) | -6.96 (-9.06 to -4.7) |
| UI, uncertainty interval. | | | | | |

| Table S9. Cases and rates per 100 000 of the population and percentage change of DALYs for age-related macular degeneration by sex and age, 1990–2021 | | | | | |
| --- | --- | --- | --- | --- | --- |
|  | 1990 | | 2021 | | 1990 to 2021 |
|  | Cases (95% UI) | Rates (95% UI) | Cases (95% UI) | Rates (95% UI) | Percentage change, % (95%UI) |
| **Global** | 333464 (221441 to 475365) | 9.28 (6.13 to 13.12) | 638116 (430431 to 903722) | 7.5 (5.05 to 10.58) | -19.19 (-22.38 to -15.69) |
| **Japan** | 4223 (2640 to 6067) | 2.6 (1.64 to 3.71) | 10003 (6540 to 14275) | 2.14 (1.38 to 3.03) | -17.74 (-21.17 to -13.38) |
| **Sex** |  |  |  |  |  |
| Male | 1708 (1046 to 2448) | 2.61 (1.62 to 3.7) | 4018 (2592 to 5787) | 2.18 (1.39 to 3.11) | -16.57 (-21.81 to -10.59) |
| Female | 2515 (1596 to 3591) | 2.59 (1.65 to 3.71) | 5985 (3906 to 8517) | 2.1 (1.35 to 2.98) | -18.88 (-23.31 to -13.76) |
| **Age** |  |  |  |  |  |
| 45-49 years | 21 (8 to 45) | 0.23 (0.09 to 0.49) | 17 (7 to 37) | 0.17 (0.07 to 0.37) | -24.76 (-35.42 to -12.53) |
| 50-54 years | 122 (58 to 212) | 1.49 (0.71 to 2.58) | 104 (50 to 182) | 1.11 (0.53 to 1.94) | -25.54 (-32.03 to -18.02) |
| 55-59 years | 287 (150 to 503) | 3.68 (1.93 to 6.45) | 223 (109 to 372) | 2.82 (1.39 to 4.72) | -23.34 (-38.26 to -5.74) |
| 60-64 years | 452 (231 to 738) | 6.65 (3.4 to 10.87) | 390 (203 to 629) | 5.23 (2.72 to 8.43) | -21.33 (-35.83 to -4.03) |
| 65-69 years | 538 (320 to 829) | 10.46 (6.22 to 16.12) | 667 (402 to 1015) | 8.39 (5.06 to 12.77) | -19.79 (-31.09 to -6.14) |
| 70-74 years | 615 (377 to 936) | 15.99 (9.8 to 24.34) | 1265 (797 to 1940) | 12.96 (8.17 to 19.88) | -18.93 (-28.31 to -7.62) |
| 75-79 years | 760 (474 to 1127) | 25.02 (15.59 to 37.09) | 1402 (876 to 2088) | 20.63 (12.9 to 30.74) | -17.55 (-24.9 to -8.02) |
| 80-84 years | 697 (429 to 1056) | 37.79 (23.27 to 57.25) | 1769 (1089 to 2685) | 31.56 (19.42 to 47.9) | -16.47 (-22.27 to -10.54) |
| 85-89 years | 463 (281 to 688) | 55.21 (33.54 to 82.07) | 1836 (1142 to 2723) | 47.26 (29.39 to 70.1) | -14.4 (-19.63 to -8.59) |
| 90-94 years | 205 (129 to 308) | 79.43 (49.77 to 119.1) | 1378 (861 to 2081) | 69.61 (43.52 to 105.14) | -12.36 (-16.52 to -7.7) |
| 95+ years | 63 (37 to 100) | 119.76 (71.16 to 190.8) | 953 (569 to 1491) | 108 (64.52 to 169.03) | -9.82 (-13.88 to -5.52) |
| DALYs, disability-adjusted life years; UI, uncertainty interval. | | | | | |

| Table S10. Cases and age-standardized rates (per 100,000 population) of DALYs for vision impairment due to AMD attributable to tobacco and temporal trends by sex and age group, 1990–2021 | | | | | |
| --- | --- | --- | --- | --- | --- |
|  | 1990 | | 2021 | | 1990 to 2021 |
|  | Cases (95% UI) | Rates (95% UI) | Cases (95% UI) | Rates (95% UI) | Percentage change, % (95%UI) |
| **Global** | 40006 (21920 to 65048) | 1.04 (0.57 to 1.7) | 58858 (31938 to 96955) | 0.68 (0.37 to 1.11) | -35.27 (-39.6 to -30.94) |
| **Japan** | 603 (323 to 976) | 0.36 (0.19 to 0.57) | 669 (337 to 1145) | 0.19 (0.1 to 0.32) | -47.23 (-54.67 to -39.89) |
| **Sex** |  |  |  |  |  |
| Male | 465 (249 to 733) | 0.66 (0.36 to 1.03) | 519 (266 to 879) | 0.32 (0.17 to 0.54) | -51.35 (-58.81 to -43.93) |
| Female | 138 (67 to 240) | 0.14 (0.07 to 0.25) | 150 (72 to 264) | 0.08 (0.04 to 0.14) | -46.36 (-59.65 to -30.69) |
| **Age** |  |  |  |  |  |
| 45-49 years | 6 (2 to 13) | 0.07 (0.02 to 0.14) | 4 (1 to 8) | 0.04 (0.01 to 0.08) | -45.06 (-57.6 to -29.39) |
| 50-54 years | 33 (14 to 65) | 0.4 (0.17 to 0.8) | 21 (9 to 41) | 0.22 (0.09 to 0.44) | -43.83 (-55.59 to -32.21) |
| 55-59 years | 70 (31 to 126) | 0.9 (0.4 to 1.61) | 41 (19 to 78) | 0.53 (0.24 to 0.99) | -41.82 (-57.46 to -21.19) |
| 60-64 years | 111 (53 to 190) | 1.63 (0.78 to 2.8) | 63 (29 to 117) | 0.84 (0.38 to 1.57) | -48.52 (-61.37 to -33.3) |
| 65-69 years | 110 (55 to 183) | 2.14 (1.07 to 3.56) | 91 (45 to 160) | 1.14 (0.56 to 2.02) | -46.63 (-60.62 to -30.39) |
| 70-74 years | 87 (44 to 149) | 2.26 (1.14 to 3.88) | 120 (57 to 221) | 1.23 (0.58 to 2.26) | -45.51 (-59.81 to -27.36) |
| 75-79 years | 76 (39 to 128) | 2.51 (1.29 to 4.22) | 94 (45 to 166) | 1.39 (0.66 to 2.44) | -44.76 (-60.55 to -27.78) |
| 80-84 years | 58 (29 to 100) | 3.16 (1.57 to 5.45) | 86 (39 to 161) | 1.54 (0.7 to 2.87) | -51.12 (-65.74 to -33.48) |
| 85-89 years | 35 (17 to 59) | 4.13 (2.04 to 6.98) | 75 (35 to 137) | 1.94 (0.9 to 3.54) | -53.05 (-66.71 to -36.29) |
| 90-94 years | 14 (7 to 23) | 5.27 (2.6 to 9.06) | 47 (22 to 91) | 2.37 (1.1 to 4.6) | -55.02 (-68.46 to -38.08) |
| 95+ years | 4 (2 to 7) | 7.08 (3.38 to 12.5) | 27 (12 to 53) | 3.04 (1.31 to 6.03) | -57.09 (-70.8 to -39.57) |
| **Prefectures** |  |  |  |  |  |
| Hokkaido | 28 (15 to 50) | 0.37 (0.19 to 0.64) | 29 (14 to 50) | 0.18 (0.08 to 0.3) | -52.27 (-66.01 to -35.84) |
| Aomori | 8 (4 to 13) | 0.35 (0.17 to 0.6) | 7 (3 to 12) | 0.17 (0.08 to 0.29) | -51.91 (-64.54 to -35.5) |
| Iwate | 8 (4 to 14) | 0.36 (0.18 to 0.59) | 7 (3 to 12) | 0.18 (0.09 to 0.31) | -50.11 (-63.26 to -29.48) |
| Miyagi | 11 (5 to 19) | 0.36 (0.17 to 0.61) | 12 (6 to 22) | 0.19 (0.1 to 0.33) | -46.57 (-61.36 to -29.55) |
| Akita | 7 (4 to 13) | 0.35 (0.17 to 0.59) | 6 (3 to 10) | 0.17 (0.08 to 0.29) | -52.55 (-65.09 to -37.34) |
| Yamagata | 8 (4 to 13) | 0.35 (0.18 to 0.57) | 6 (3 to 11) | 0.17 (0.08 to 0.31) | -50.5 (-64.56 to -33) |
| Fukushima | 12 (6 to 20) | 0.36 (0.19 to 0.6) | 11 (5 to 19) | 0.19 (0.09 to 0.33) | -47.93 (-62.73 to -31.21) |
| Ibaraki | 14 (7 to 23) | 0.37 (0.19 to 0.61) | 16 (8 to 29) | 0.2 (0.1 to 0.34) | -46.59 (-60.2 to -28.91) |
| Tochigi | 10 (5 to 16) | 0.37 (0.18 to 0.59) | 11 (6 to 19) | 0.2 (0.1 to 0.35) | -44.87 (-58.21 to -28.49) |
| Gunma | 10 (5 to 17) | 0.36 (0.18 to 0.59) | 11 (5 to 18) | 0.19 (0.09 to 0.32) | -47.35 (-60.93 to -29.52) |
| Saitama | 24 (12 to 40) | 0.38 (0.19 to 0.62) | 40 (20 to 70) | 0.2 (0.1 to 0.36) | -45.6 (-59.69 to -27.88) |
| Chiba | 23 (12 to 37) | 0.37 (0.19 to 0.61) | 34 (17 to 61) | 0.2 (0.1 to 0.34) | -45.8 (-59.97 to -28.56) |
| Tokyo | 53 (28 to 91) | 0.37 (0.2 to 0.62) | 68 (35 to 113) | 0.2 (0.1 to 0.35) | -44.19 (-58.86 to -26.77) |
| Kanagawa | 32 (16 to 54) | 0.38 (0.19 to 0.62) | 49 (25 to 82) | 0.2 (0.1 to 0.35) | -45.7 (-60.84 to -27.86) |
| Niigata | 14 (7 to 24) | 0.34 (0.17 to 0.58) | 12 (6 to 22) | 0.18 (0.09 to 0.31) | -48.07 (-61.73 to -31.75) |
| Toyama | 6 (3 to 11) | 0.34 (0.18 to 0.57) | 6 (3 to 10) | 0.17 (0.08 to 0.31) | -49.1 (-62.67 to -31.82) |
| Ishikawa | 6 (3 to 10) | 0.34 (0.18 to 0.57) | 6 (3 to 10) | 0.18 (0.09 to 0.31) | -46.87 (-60.58 to -28.19) |
| Fukui | 5 (2 to 8) | 0.35 (0.18 to 0.58) | 4 (2 to 7) | 0.18 (0.08 to 0.31) | -49.34 (-62.48 to -31.38) |
| Yamanashi | 5 (3 to 8) | 0.36 (0.19 to 0.6) | 5 (2 to 8) | 0.18 (0.09 to 0.32) | -49.06 (-63.2 to -31.63) |
| Nagano | 13 (7 to 21) | 0.34 (0.18 to 0.56) | 12 (6 to 22) | 0.18 (0.09 to 0.32) | -48.25 (-63.3 to -32.27) |
| Gifu | 10 (5 to 16) | 0.33 (0.17 to 0.54) | 10 (5 to 19) | 0.18 (0.08 to 0.31) | -46.52 (-60.25 to -29.89) |
| Shizuoka | 18 (9 to 31) | 0.36 (0.18 to 0.6) | 20 (9 to 36) | 0.19 (0.09 to 0.34) | -47.83 (-62.9 to -29.47) |
| Aichi | 28 (14 to 46) | 0.36 (0.19 to 0.59) | 38 (18 to 65) | 0.2 (0.09 to 0.34) | -45.69 (-60.87 to -26.4) |
| Mie | 10 (5 to 16) | 0.35 (0.18 to 0.58) | 10 (5 to 17) | 0.18 (0.09 to 0.32) | -47.81 (-61.61 to -28.91) |
| Shiga | 6 (3 to 9) | 0.35 (0.18 to 0.57) | 7 (4 to 12) | 0.19 (0.1 to 0.33) | -45.02 (-60.59 to -27.46) |
| Kyoto | 13 (6 to 21) | 0.35 (0.17 to 0.58) | 13 (6 to 23) | 0.18 (0.09 to 0.31) | -48.55 (-62.5 to -31.06) |
| Osaka | 37 (20 to 62) | 0.37 (0.2 to 0.6) | 45 (23 to 80) | 0.19 (0.1 to 0.33) | -48.43 (-61.74 to -32.49) |
| Hyogo | 26 (13 to 44) | 0.36 (0.18 to 0.6) | 28 (14 to 49) | 0.18 (0.09 to 0.31) | -49.43 (-62.19 to -31.29) |
| Nara | 6 (3 to 11) | 0.35 (0.18 to 0.59) | 7 (3 to 12) | 0.17 (0.08 to 0.3) | -51.22 (-64.14 to -35) |
| Wakayama | 6 (3 to 11) | 0.35 (0.18 to 0.58) | 5 (2 to 9) | 0.17 (0.08 to 0.3) | -51.44 (-64.67 to -34.62) |
| Tottori | 4 (2 to 6) | 0.34 (0.17 to 0.57) | 3 (2 to 5) | 0.18 (0.08 to 0.32) | -48.1 (-62.95 to -28.27) |
| Shimane | 5 (3 to 9) | 0.34 (0.17 to 0.56) | 4 (2 to 7) | 0.17 (0.08 to 0.29) | -50.38 (-64.49 to -33.1) |
| Okayama | 11 (5 to 18) | 0.35 (0.18 to 0.56) | 10 (5 to 18) | 0.18 (0.08 to 0.3) | -49.17 (-62.4 to -31.32) |
| Hiroshima | 15 (8 to 23) | 0.35 (0.19 to 0.56) | 15 (7 to 26) | 0.18 (0.09 to 0.32) | -47.47 (-61.53 to -30.12) |
| Yamaguchi | 9 (5 to 16) | 0.34 (0.17 to 0.57) | 7 (3 to 13) | 0.17 (0.08 to 0.3) | -50.9 (-65.41 to -31.75) |
| Tokushima | 5 (3 to 8) | 0.35 (0.18 to 0.58) | 4 (2 to 8) | 0.17 (0.09 to 0.31) | -50.1 (-63.37 to -30.96) |
| Kagawa | 6 (3 to 10) | 0.35 (0.18 to 0.56) | 5 (3 to 10) | 0.18 (0.09 to 0.32) | -47.77 (-62.5 to -31.5) |
| Ehime | 9 (5 to 14) | 0.34 (0.18 to 0.56) | 7 (4 to 13) | 0.17 (0.09 to 0.29) | -50.18 (-63 to -33.53) |
| Kochi | 5 (3 to 9) | 0.35 (0.17 to 0.58) | 4 (2 to 7) | 0.17 (0.08 to 0.29) | -51.33 (-64.48 to -36.53) |
| Fukuoka | 23 (12 to 40) | 0.34 (0.18 to 0.58) | 25 (13 to 44) | 0.18 (0.09 to 0.31) | -48.01 (-61.92 to -31.27) |
| Saga | 5 (2 to 8) | 0.34 (0.17 to 0.56) | 4 (2 to 8) | 0.17 (0.08 to 0.3) | -48.95 (-62.53 to -31.28) |
| Nagasaki | 9 (4 to 15) | 0.34 (0.17 to 0.59) | 7 (3 to 12) | 0.17 (0.08 to 0.28) | -50.42 (-63.88 to -33.42) |
| Kumamoto | 10 (5 to 17) | 0.34 (0.17 to 0.55) | 9 (5 to 16) | 0.17 (0.09 to 0.31) | -49.11 (-63.03 to -31.29) |
| Oita | 7 (4 to 12) | 0.34 (0.18 to 0.57) | 6 (3 to 11) | 0.17 (0.08 to 0.3) | -50.79 (-63.26 to -33.7) |
| Miyazaki | 6 (3 to 10) | 0.34 (0.18 to 0.56) | 6 (3 to 10) | 0.17 (0.08 to 0.3) | -50.88 (-64.12 to -34.21) |
| Kagoshima | 11 (5 to 17) | 0.34 (0.17 to 0.54) | 9 (4 to 15) | 0.17 (0.08 to 0.29) | -50.27 (-63.39 to -36.31) |
| Okinawa | 5 (2 to 8) | 0.34 (0.17 to 0.54) | 8 (4 to 13) | 0.22 (0.11 to 0.38) | -34.92 (-52.37 to -13.09) |
| DALYs, disability-adjusted life years; UI, uncertainty interval. | | | | | |

| Table S11. Age-standardized percentage and percentage change of DALY attributed to tobacco of vision impairment due to age-related macular degeneration by sex and age, 1990–2021 | | | | | | | | | |
| --- | --- | --- | --- | --- | --- | --- | --- | --- | --- |
|  | Percentage (%) | | | | | | | | |
|  | 1990 (95% UI) | | | 2021 (95% UI) | | | Percentage change between 1990 to 2021 (95% UI) | | |
|  | Both | Male | Female | Both | Male | Female | Both | Male | Female |
| **Global** | 12.45 (7.73 to 17.37) | 22.61 (14.29 to 30.61) | 6.02 (3.47 to 8.73) | 9.96 (6.12 to 14.06) | 18.72 (11.69 to 26.02) | 3.89 (2.2 to 5.83) | -20.03 (-23.43 to -17.19) | -17.21 (-20.36 to -14.35) | -35.35 (-40.34 to -29.79) |
| **Japan** | 15.19 (9.41 to 21.25) | 28.25 (17.98 to 38.57) | 6.15 (3.38 to 9.37) | 9.68 (5.57 to 14.34) | 16.35 (9.71 to 23.99) | 4.04 (2.13 to 6.32) | -36.3 (-44.33 to -28.36) | -42.12 (-50.25 to -34.48) | -34.29 (-49.81 to -14.86) |
| **Age** |  |  |  |  |  |  |  |  |  |
| 45-49 years | 28.31 (18.38 to 38.03) | 40.38 (26.99 to 52.42) | 12.73 (7.15 to 19.1) | 20.67 (12.38 to 29.94) | 28.35 (16.94 to 40.76) | 10.16 (4.63 to 18.3) | -27 (-40.13 to -11.7) | -29.78 (-42.7 to -15.99) | -20.18 (-53.82 to 27.73) |
| 50-54 years | 26.77 (17.35 to 36.18) | 40.7 (27.09 to 53.28) | 10.49 (5.87 to 16.47) | 20.17 (12.45 to 28.75) | 29.38 (17.8 to 40.54) | 8.49 (3.85 to 15.41) | -24.63 (-37.11 to -10.81) | -27.82 (-40.86 to -14.24) | -19.09 (-52.82 to 29.85) |
| 55-59 years | 25.73 (16.57 to 34.87) | 39.43 (26.34 to 51.48) | 11.25 (6.17 to 17.42) | 19.36 (11.25 to 27.84) | 28.38 (17.21 to 39.86) | 9.08 (3.94 to 15.95) | -24.73 (-39.97 to -9.25) | -28.02 (-41.14 to -13.49) | -19.31 (-52.34 to 29.7) |
| 60-64 years | 26.22 (16.95 to 35.24) | 41.81 (28.32 to 53.88) | 11.11 (6.02 to 17.55) | 17 (9.68 to 25.56) | 26.52 (15.84 to 38.14) | 6.7 (3.06 to 12.02) | -35.17 (-49.06 to -19.03) | -36.56 (-50.49 to -22.45) | -39.71 (-66.42 to 6.2) |
| 65-69 years | 22.21 (13.6 to 30.91) | 35.7 (23.44 to 47.47) | 12.3 (6.6 to 19.25) | 14.64 (8.54 to 21.67) | 21.01 (12.23 to 31.02) | 8.4 (4.1 to 14.74) | -34.08 (-49.01 to -15.18) | -41.15 (-54.02 to -26.61) | -31.7 (-62.07 to 19.53) |
| 70-74 years | 15.76 (9.25 to 22.81) | 26.77 (16.65 to 36.63) | 8.29 (3.96 to 13.24) | 10.43 (5.75 to 16.42) | 16.07 (9.08 to 24.63) | 5.19 (2.17 to 9.71) | -33.8 (-49.72 to -13.55) | -39.98 (-55.75 to -22.14) | -37.45 (-65.62 to 4.62) |
| 75-79 years | 11.27 (6.9 to 16.31) | 26.32 (16.18 to 37.43) | 1.56 (0.67 to 2.92) | 7.48 (4.21 to 11.47) | 14.74 (8.38 to 22.49) | 1.45 (0.61 to 2.74) | -33.62 (-51.2 to -13.93) | -43.97 (-58.27 to -28.32) | -7.26 (-55.56 to 88.38) |
| 80-84 years | 9.47 (5.52 to 13.77) | 20.84 (12.18 to 30) | 2.9 (1.44 to 5.09) | 5.5 (2.77 to 8.78) | 10.55 (5.43 to 16.92) | 1.87 (0.78 to 3.52) | -41.92 (-57.63 to -20.98) | -49.4 (-64.64 to -31.32) | -35.6 (-67.85 to 21.22) |
| 85-89 years | 8.59 (5.02 to 12.59) | 20.39 (11.89 to 29.36) | 2.79 (1.39 to 4.93) | 4.69 (2.32 to 7.62) | 9.94 (4.99 to 16.34) | 1.72 (0.7 to 3.38) | -45.38 (-60.79 to -25.57) | -51.24 (-66.49 to -33.37) | -38.17 (-70.95 to 19.31) |
| 90-94 years | 7.73 (4.47 to 11.3) | 20.01 (11.74 to 28.84) | 2.71 (1.34 to 4.85) | 3.94 (1.92 to 6.41) | 9.47 (4.7 to 15.88) | 1.62 (0.64 to 3.23) | -49 (-64.51 to -29.12) | -52.68 (-67.71 to -34.21) | -40.25 (-72.93 to 17.45) |
| 95+ years | 6.95 (4.04 to 10.27) | 19.42 (11.44 to 28.18) | 2.58 (1.27 to 4.72) | 3.28 (1.61 to 5.45) | 8.79 (4.32 to 14.9) | 1.46 (0.53 to 3.03) | -52.76 (-67.97 to -33.6) | -54.7 (-69.59 to -36.7) | -43.43 (-76.88 to 16.27) |
| **Prefectures** |  |  |  |  |  |  |  |  |  |
| Hokkaido | 15.45 (9.38 to 22.08) | 28.09 (17.49 to 38.96) | 6.11 (3.01 to 9.94) | 8.95 (5.01 to 13.6) | 15.44 (9.03 to 22.83) | 3.82 (1.82 to 6.26) | -42.05 (-54.95 to -27.25) | -45.03 (-56.87 to -32.54) | -37.5 (-61.4 to 1.05) |
| Aomori | 14.55 (8.47 to 20.8) | 27.49 (16.61 to 38.42) | 5.96 (2.95 to 10.07) | 8.61 (5.05 to 13.1) | 14.83 (8.49 to 22.19) | 3.67 (1.81 to 6.29) | -40.78 (-52.81 to -25.98) | -46.05 (-56.72 to -33.41) | -38.4 (-62.28 to 2.63) |
| Iwate | 14.88 (8.46 to 21.27) | 27.75 (17.08 to 38.23) | 6.02 (2.98 to 10.41) | 9.08 (5.14 to 13.72) | 15.39 (8.82 to 22.92) | 3.83 (1.83 to 6.54) | -39.02 (-53.37 to -21.94) | -44.54 (-56.5 to -30.36) | -36.34 (-61.98 to 8.62) |
| Miyagi | 15.25 (9.12 to 21.91) | 28.23 (17.71 to 39.43) | 6.22 (3.07 to 10.39) | 9.91 (5.7 to 14.98) | 16.64 (9.55 to 24.48) | 4.13 (2.05 to 7.21) | -35.01 (-49.17 to -17.96) | -41.04 (-52.37 to -28.1) | -33.63 (-59.86 to 13.82) |
| Akita | 14.67 (8.64 to 20.8) | 27.77 (16.86 to 38.27) | 6.01 (2.85 to 9.88) | 8.37 (4.71 to 13.13) | 14.27 (8.21 to 21) | 3.62 (1.77 to 6.47) | -42.96 (-54.99 to -28.99) | -48.59 (-59.22 to -36.46) | -39.77 (-62.91 to -1.11) |
| Yamagata | 14.89 (8.91 to 21.42) | 27.86 (17.08 to 39) | 6.06 (3.11 to 9.94) | 8.83 (4.86 to 13.87) | 14.81 (8.56 to 22.21) | 3.72 (1.82 to 6.46) | -40.67 (-53.97 to -25.82) | -46.83 (-58.15 to -34.43) | -38.71 (-61 to -2.97) |
| Fukushima | 15.09 (9.4 to 21.49) | 28.02 (17.76 to 38.82) | 6.15 (3.21 to 10.57) | 9.57 (5.36 to 14.59) | 16.02 (9.15 to 23.77) | 3.97 (1.85 to 6.75) | -36.61 (-51.38 to -20.85) | -42.82 (-55.56 to -30.49) | -35.36 (-59.54 to 1.42) |
| Ibaraki | 15.53 (9.75 to 21.99) | 28.57 (18.28 to 38.72) | 6.2 (3.2 to 10.25) | 10.09 (5.44 to 15.14) | 16.68 (9.44 to 24.56) | 4.14 (2.01 to 7.05) | -35.02 (-48.65 to -18.21) | -41.6 (-53.66 to -27.96) | -33.25 (-58.96 to 9.51) |
| Tochigi | 15.38 (9.29 to 21.93) | 28.53 (17.78 to 39.04) | 6.23 (3.18 to 10.31) | 10.23 (6.03 to 15.59) | 17.06 (9.95 to 25.34) | 4.22 (2.13 to 7.36) | -33.5 (-46.4 to -18.28) | -40.19 (-52 to -26.39) | -32.3 (-58.31 to 12.15) |
| Gunma | 15.5 (9.21 to 22) | 28.35 (17.43 to 39.02) | 6.2 (3.05 to 10.25) | 9.82 (5.62 to 14.77) | 16.34 (9.43 to 24.36) | 4.02 (1.87 to 7.06) | -36.63 (-50.31 to -21.02) | -42.37 (-54.25 to -30.02) | -35.17 (-62.85 to 2.66) |
| Saitama | 16 (9.89 to 22.98) | 29.3 (18.54 to 40.61) | 6.48 (3.31 to 10.37) | 10.56 (6.12 to 16.12) | 17.59 (10.51 to 26.55) | 4.35 (2.15 to 7.82) | -34.02 (-47.75 to -16.1) | -39.96 (-51.37 to -26.08) | -32.87 (-57.93 to 5.97) |
| Chiba | 15.86 (9.67 to 22.33) | 29.13 (18.36 to 39.99) | 6.43 (3.29 to 10.56) | 10.34 (5.97 to 15.96) | 17.24 (10.26 to 25.83) | 4.19 (2.08 to 7.13) | -34.77 (-48.35 to -19.86) | -40.82 (-51.21 to -28.24) | -34.77 (-60.91 to 5.36) |
| Tokyo | 15.99 (9.67 to 23.11) | 29.53 (18.71 to 40.01) | 6.49 (3.22 to 10.71) | 10.69 (6.3 to 15.93) | 18.16 (10.66 to 26.68) | 4.4 (2.25 to 7.42) | -33.15 (-46.57 to -16.1) | -38.5 (-49.74 to -26.19) | -32.11 (-55.72 to 7.73) |
| Kanagawa | 16.39 (10.1 to 23.06) | 29.85 (19.17 to 40.52) | 6.65 (3.52 to 10.95) | 10.61 (6.16 to 15.86) | 17.71 (10.4 to 25.72) | 4.36 (2.16 to 7.52) | -35.24 (-49.33 to -20.2) | -40.66 (-53.1 to -27.41) | -34.44 (-59.72 to 9.57) |
| Niigata | 14.93 (8.87 to 21.16) | 27.97 (17.42 to 39.33) | 6.04 (3 to 10.19) | 9.21 (5.11 to 13.74) | 15.51 (8.74 to 23.63) | 3.88 (1.89 to 6.87) | -38.33 (-51.24 to -21.91) | -44.55 (-55.61 to -32.29) | -35.75 (-60.73 to 5.51) |
| Toyama | 14.69 (8.82 to 21.33) | 27.62 (17.59 to 38.23) | 5.95 (2.92 to 9.63) | 9.01 (4.97 to 13.83) | 15.33 (8.56 to 22.87) | 3.81 (1.81 to 6.87) | -38.63 (-51.96 to -21.37) | -44.49 (-56.19 to -30.35) | -36.08 (-59.57 to 5.55) |
| Ishikawa | 14.74 (8.92 to 20.97) | 27.65 (17.29 to 37.89) | 5.94 (3.19 to 9.55) | 9.41 (5.24 to 14.62) | 15.9 (9.11 to 23.61) | 3.96 (2.01 to 6.68) | -36.19 (-50.04 to -20.24) | -42.49 (-54.38 to -29.15) | -33.36 (-59.47 to 5.79) |
| Fukui | 15.01 (9.17 to 21.05) | 27.84 (17.47 to 38.46) | 6.05 (3.05 to 10.19) | 9.18 (5.2 to 14) | 15.41 (8.66 to 23.59) | 3.86 (1.75 to 6.79) | -38.87 (-51.61 to -22.92) | -44.62 (-56.58 to -32.86) | -36.26 (-62.28 to -0.62) |
| Yamanashi | 15.34 (9.16 to 21.69) | 28.33 (17.68 to 38.85) | 6.14 (3.25 to 9.66) | 9.46 (5.37 to 13.97) | 15.82 (8.79 to 22.77) | 3.89 (1.87 to 6.7) | -38.34 (-52.24 to -20.33) | -44.16 (-55.55 to -30.93) | -36.64 (-61.77 to 2.53) |
| Nagano | 14.96 (8.93 to 21.69) | 27.69 (17.4 to 38.67) | 6 (3.07 to 9.9) | 9.19 (5.29 to 14.21) | 15.24 (8.85 to 22.85) | 3.84 (1.84 to 6.65) | -38.56 (-52.35 to -23.15) | -44.96 (-56.96 to -32.6) | -35.98 (-60.41 to 8.92) |
| Gifu | 15.19 (9.15 to 21.42) | 27.81 (17.28 to 38.12) | 6.12 (3.13 to 9.91) | 9.14 (5.26 to 13.81) | 15.64 (9.17 to 23.08) | 3.95 (1.91 to 6.72) | -39.84 (-52.53 to -23.33) | -43.75 (-54.6 to -31.41) | -35.45 (-61.15 to 8.1) |
| Shizuoka | 15.32 (9.42 to 21.52) | 28.51 (17.41 to 39.37) | 6.13 (3.21 to 10.49) | 9.66 (5.49 to 15.2) | 16.16 (9.35 to 24.89) | 4.02 (1.91 to 6.76) | -36.91 (-51.08 to -21.08) | -43.32 (-54.92 to -29.78) | -34.48 (-61.17 to 9.05) |
| Aichi | 15.73 (9.47 to 22.31) | 28.72 (17.99 to 39.22) | 6.22 (3.33 to 10.01) | 10.41 (5.79 to 15.73) | 17.35 (9.95 to 26.23) | 4.23 (2.34 to 7.33) | -33.85 (-49.1 to -17.3) | -39.59 (-51.64 to -26.2) | -31.97 (-59.71 to 9.69) |
| Mie | 14.95 (9.24 to 21.75) | 27.88 (17.55 to 38.76) | 6.1 (2.97 to 10.39) | 9.46 (5.29 to 14.51) | 15.9 (9.15 to 23.97) | 3.94 (2.03 to 6.77) | -36.71 (-51.38 to -18.82) | -42.98 (-55 to -30.59) | -35.45 (-61.52 to 10.74) |
| Shiga | 14.96 (9.17 to 21.56) | 28.04 (17.21 to 38.1) | 6.04 (2.98 to 10.01) | 9.93 (5.69 to 14.99) | 16.67 (9.37 to 24.24) | 4.13 (2.04 to 7.25) | -33.58 (-47.54 to -17.83) | -40.56 (-51.57 to -26.96) | -31.63 (-58.4 to 10.67) |
| Kyoto | 14.92 (9.2 to 21.63) | 27.93 (17.69 to 38.72) | 6.04 (3.08 to 9.93) | 9.32 (5.27 to 13.81) | 15.81 (8.96 to 23.63) | 3.97 (1.92 to 6.58) | -37.55 (-50.86 to -19.99) | -43.41 (-54.84 to -31.15) | -34.32 (-59.43 to 4.6) |
| Osaka | 15.38 (9.37 to 21.68) | 28.89 (18.13 to 39.84) | 6.25 (3.19 to 10.11) | 9.59 (5.57 to 14.7) | 16.37 (9.82 to 24.36) | 4.04 (1.92 to 6.82) | -37.62 (-50.95 to -22.72) | -43.34 (-54.02 to -31.37) | -35.27 (-61.44 to 6.54) |
| Hyogo | 15.07 (8.91 to 21.56) | 28.18 (17.4 to 39.01) | 6.15 (3.14 to 10.19) | 9.29 (5.17 to 14.31) | 15.8 (9.14 to 23.46) | 3.93 (1.9 to 6.19) | -38.32 (-51.87 to -21.88) | -43.92 (-54.84 to -31.92) | -36.17 (-59.92 to 4.15) |
| Nara | 15.15 (8.93 to 21.65) | 27.98 (17.06 to 38.6) | 6.02 (3.08 to 9.97) | 8.92 (4.91 to 13.55) | 15.15 (8.61 to 22.13) | 3.81 (1.85 to 6.66) | -41.12 (-55.47 to -26.45) | -45.85 (-57.27 to -32.97) | -36.62 (-62.48 to 4.59) |
| Wakayama | 14.61 (9.13 to 20.76) | 27.43 (17.07 to 38.23) | 5.92 (2.99 to 9.64) | 8.55 (4.88 to 13.26) | 14.59 (8.38 to 21.55) | 3.67 (1.79 to 6.42) | -41.44 (-53.91 to -27.44) | -46.83 (-57.53 to -34.9) | -38 (-62.85 to 2.32) |
| Tottori | 14.39 (8.68 to 20.21) | 27.47 (16.86 to 37.59) | 5.91 (2.95 to 9.66) | 8.98 (5.01 to 13.64) | 15.29 (8.8 to 22.5) | 3.81 (1.85 to 6.63) | -37.59 (-51.46 to -21.3) | -44.34 (-57.21 to -31.38) | -35.62 (-60.38 to 2.23) |
| Shimane | 14.4 (8.47 to 20.63) | 27.1 (16.79 to 37.74) | 5.85 (2.82 to 9.9) | 8.63 (5.08 to 13.42) | 14.48 (8.54 to 22.03) | 3.67 (1.76 to 6.64) | -40.03 (-53.04 to -25.28) | -46.55 (-57.23 to -34.91) | -37.31 (-62.58 to 4.02) |
| Okayama | 14.77 (8.85 to 21.07) | 27.46 (17.2 to 38.65) | 5.92 (3.18 to 9.7) | 9.1 (5.06 to 13.66) | 15.38 (8.8 to 22.96) | 3.86 (1.88 to 6.87) | -38.42 (-52.22 to -23.14) | -44.01 (-55.52 to -31.55) | -34.82 (-62.82 to 2.43) |
| Hiroshima | 15.05 (9.04 to 21.6) | 27.84 (17.56 to 38.32) | 6 (3.14 to 10.19) | 9.46 (5.45 to 14.24) | 16.02 (9.3 to 23.79) | 3.96 (2.03 to 6.83) | -37.15 (-51.78 to -19.96) | -42.43 (-54.35 to -29.69) | -34 (-59.67 to 11.8) |
| Yamaguchi | 14.39 (8.38 to 20.62) | 27.13 (16.98 to 37.45) | 5.9 (2.94 to 9.77) | 8.5 (4.83 to 13.07) | 14.59 (8.31 to 22.12) | 3.64 (1.7 to 6.24) | -40.95 (-54.7 to -25.17) | -46.22 (-57.43 to -33.77) | -38.29 (-62.56 to -2.09) |
| Tokushima | 14.74 (8.68 to 20.92) | 27.55 (17.21 to 37.98) | 5.96 (2.92 to 9.91) | 8.85 (4.93 to 13.47) | 14.95 (8.28 to 22.45) | 3.77 (1.85 to 6.62) | -39.95 (-54.37 to -23.46) | -45.72 (-57.74 to -33.38) | -36.79 (-62.47 to 0.58) |
| Kagawa | 14.72 (8.95 to 20.39) | 27.45 (17.33 to 37.84) | 5.93 (3.07 to 9.8) | 9.21 (5.14 to 13.88) | 15.54 (8.95 to 23.13) | 3.85 (1.94 to 6.61) | -37.45 (-52.42 to -22.17) | -43.41 (-55.8 to -30.39) | -35.08 (-60.44 to 7.73) |
| Ehime | 14.57 (8.75 to 20.24) | 27.22 (16.45 to 37.22) | 5.87 (2.83 to 9.65) | 8.73 (4.93 to 13.34) | 14.79 (8.55 to 22.23) | 3.72 (1.86 to 6.29) | -40.07 (-53.6 to -25.91) | -45.69 (-57.06 to -33.5) | -36.59 (-61.02 to 3.65) |
| Kochi | 14.38 (8.9 to 20.56) | 27.12 (17.16 to 37.66) | 5.92 (2.98 to 9.71) | 8.52 (4.89 to 13.37) | 14.48 (8.35 to 21.94) | 3.63 (1.68 to 6.47) | -40.73 (-53.81 to -26.28) | -46.59 (-57.98 to -34.47) | -38.59 (-64 to 0.7) |
| Fukuoka | 14.63 (8.8 to 21.51) | 27.71 (17.2 to 37.84) | 5.99 (3.13 to 9.98) | 9.25 (5.18 to 14.19) | 15.9 (9.2 to 23.83) | 3.97 (1.91 to 6.83) | -36.76 (-50.76 to -21.47) | -42.6 (-54.82 to -29.42) | -33.72 (-58.27 to 4.89) |
| Saga | 14.1 (8.18 to 20.69) | 26.84 (16.66 to 37.33) | 5.8 (2.79 to 9.64) | 8.77 (4.98 to 13.27) | 15 (8.34 to 22.67) | 3.73 (1.79 to 6.54) | -37.77 (-51.7 to -21.26) | -44.11 (-56.14 to -30.43) | -35.71 (-62.28 to 5.82) |
| Nagasaki | 14.11 (8.59 to 20.5) | 27.1 (16.98 to 37.88) | 5.8 (2.7 to 9.74) | 8.56 (4.74 to 13.05) | 14.7 (8.38 to 22.24) | 3.66 (1.65 to 6.27) | -39.33 (-52.81 to -22.26) | -45.76 (-56.17 to -33.27) | -36.97 (-60.34 to 3.77) |
| Kumamoto | 14.27 (8.6 to 20.85) | 27.13 (16.94 to 37.7) | 5.84 (2.88 to 9.54) | 8.78 (5.08 to 13.53) | 14.91 (8.58 to 22.81) | 3.73 (1.76 to 6.5) | -38.5 (-52.1 to -21.98) | -45.02 (-57.12 to -30.65) | -36.1 (-62.4 to 2.71) |
| Oita | 14.43 (8.57 to 20.83) | 27.04 (16.93 to 38.48) | 5.83 (2.95 to 9.64) | 8.64 (4.75 to 13.58) | 14.76 (8.33 to 21.91) | 3.73 (1.76 to 6.51) | -40.1 (-53.34 to -23.98) | -45.42 (-57.77 to -33.89) | -36.06 (-62.77 to 8.67) |
| Miyazaki | 14.29 (8.51 to 20.47) | 27.07 (16.73 to 37.59) | 5.8 (3.08 to 9.43) | 8.53 (4.75 to 13.26) | 14.52 (8.22 to 22.79) | 3.66 (1.8 to 6.39) | -40.3 (-53.59 to -24.55) | -46.35 (-57.97 to -33.25) | -36.93 (-60.88 to 3.74) |
| Kagoshima | 14.01 (8.12 to 19.92) | 26.8 (16.95 to 37.29) | 5.74 (2.81 to 9.33) | 8.52 (4.82 to 12.88) | 14.47 (8.32 to 22.11) | 3.6 (1.8 to 6.29) | -39.19 (-52.34 to -24.03) | -46 (-56.8 to -34.36) | -37.22 (-62.72 to -0.21) |
| Okinawa | 14.44 (8.63 to 20.94) | 27.81 (17.43 to 38.93) | 6.01 (3.01 to 10.11) | 10.47 (5.71 to 15.81) | 17.26 (9.6 to 25.41) | 4.25 (1.99 to 7.17) | -27.54 (-43.55 to -9.36) | -37.94 (-51.09 to -23.43) | -29.33 (-58.23 to 13.22) |
| DALYs, disability-adjusted life years; UI, uncertainty interval. | | | | | | | | | |

| Table S12. Cases and rates (per 100,000 population) of DALYs for vision impairment due to AMD attributable to tobacco, 1990–2021 | | | | | | |
| --- | --- | --- | --- | --- | --- | --- |
|  | Prevalence | | | | | |
|  | Overall (95% UI) | | Male (95% UI) | | Female (95% UI) | |
|  | Cases | Age-standardized rate (per 100,000) | Cases | Age-standardized rate (per 100,000) | Cases | Age-standardized rate (per 100,000) |
| 1990 | 603 (323 to 976) | 0.36 (0.19 to 0.57) | 465 (249 to 733) | 0.66 (0.36 to 1.03) | 138 (67 to 240) | 0.14 (0.07 to 0.25) |
| 1991 | 612 (315 to 988) | 0.35 (0.18 to 0.56) | 474 (247 to 754) | 0.65 (0.34 to 1.03) | 138 (68 to 241) | 0.14 (0.07 to 0.24) |
| 1992 | 619 (319 to 1004) | 0.34 (0.18 to 0.55) | 481 (250 to 776) | 0.63 (0.34 to 1.02) | 138 (67 to 239) | 0.13 (0.06 to 0.23) |
| 1993 | 626 (327 to 1024) | 0.33 (0.17 to 0.54) | 488 (256 to 784) | 0.62 (0.33 to 1.00) | 139 (67 to 236) | 0.13 (0.06 to 0.22) |
| 1994 | 631 (328 to 1034) | 0.32 (0.17 to 0.53) | 492 (256 to 798) | 0.61 (0.33 to 0.99) | 139 (68 to 244) | 0.13 (0.06 to 0.22) |
| 1995 | 636 (334 to 1038) | 0.32 (0.17 to 0.52) | 497 (263 to 798) | 0.59 (0.32 to 0.95) | 139 (68 to 243) | 0.12 (0.06 to 0.22) |
| 1996 | 643 (334 to 1060) | 0.31 (0.16 to 0.51) | 502 (265 to 812) | 0.58 (0.31 to 0.93) | 140 (68 to 243) | 0.12 (0.06 to 0.21) |
| 1997 | 651 (335 to 1056) | 0.30 (0.16 to 0.49) | 509 (260 to 817) | 0.57 (0.30 to 0.91) | 141 (70 to 247) | 0.12 (0.06 to 0.20) |
| 1998 | 656 (340 to 1066) | 0.30 (0.15 to 0.48) | 514 (270 to 828) | 0.55 (0.29 to 0.89) | 142 (70 to 248) | 0.12 (0.06 to 0.20) |
| 1999 | 662 (340 to 1096) | 0.29 (0.15 to 0.48) | 519 (269 to 848) | 0.54 (0.28 to 0.88) | 143 (71 to 252) | 0.11 (0.06 to 0.20) |
| 2000 | 666 (345 to 1105) | 0.28 (0.15 to 0.47) | 521 (272 to 856) | 0.53 (0.27 to 0.86) | 145 (72 to 253) | 0.11 (0.05 to 0.19) |
| 2001 | 670 (345 to 1083) | 0.28 (0.14 to 0.45) | 524 (271 to 833) | 0.51 (0.26 to 0.83) | 146 (72 to 256) | 0.11 (0.05 to 0.19) |
| 2002 | 674 (349 to 1111) | 0.27 (0.14 to 0.45) | 525 (272 to 857) | 0.50 (0.25 to 0.80) | 148 (74 to 261) | 0.11 (0.05 to 0.19) |
| 2003 | 676 (351 to 1114) | 0.26 (0.14 to 0.43) | 525 (275 to 851) | 0.48 (0.25 to 0.78) | 150 (74 to 265) | 0.11 (0.05 to 0.19) |
| 2004 | 676 (355 to 1112) | 0.26 (0.13 to 0.42) | 524 (278 to 861) | 0.46 (0.24 to 0.75) | 152 (76 to 260) | 0.11 (0.05 to 0.18) |
| 2005 | 675 (356 to 1121) | 0.25 (0.13 to 0.42) | 521 (277 to 849) | 0.45 (0.24 to 0.74) | 154 (76 to 264) | 0.10 (0.05 to 0.18) |
| 2006 | 672 (354 to 1111) | 0.24 (0.13 to 0.41) | 518 (273 to 850) | 0.43 (0.23 to 0.71) | 155 (77 to 262) | 0.10 (0.05 to 0.18) |
| 2007 | 671 (348 to 1104) | 0.24 (0.12 to 0.39) | 516 (271 to 848) | 0.42 (0.22 to 0.69) | 155 (78 to 267) | 0.10 (0.05 to 0.17) |
| 2008 | 667 (348 to 1103) | 0.23 (0.12 to 0.39) | 513 (269 to 842) | 0.41 (0.21 to 0.67) | 154 (78 to 263) | 0.10 (0.05 to 0.17) |
| 2009 | 663 (351 to 1088) | 0.22 (0.12 to 0.37) | 509 (271 to 847) | 0.39 (0.21 to 0.65) | 154 (77 to 265) | 0.10 (0.05 to 0.17) |
| 2010 | 661 (349 to 1099) | 0.22 (0.11 to 0.37) | 508 (269 to 847) | 0.38 (0.20 to 0.64) | 153 (77 to 267) | 0.09 (0.05 to 0.16) |
| 2011 | 657 (346 to 1095) | 0.21 (0.11 to 0.36) | 505 (265 to 835) | 0.37 (0.19 to 0.61) | 152 (75 to 262) | 0.09 (0.05 to 0.16) |
| 2012 | 654 (349 to 1098) | 0.21 (0.11 to 0.35) | 502 (268 to 844) | 0.36 (0.19 to 0.61) | 152 (76 to 266) | 0.09 (0.05 to 0.16) |
| 2013 | 651 (344 to 1104) | 0.21 (0.11 to 0.34) | 498 (262 to 838) | 0.35 (0.19 to 0.59) | 153 (76 to 267) | 0.09 (0.04 to 0.15) |
| 2014 | 649 (343 to 1094) | 0.20 (0.11 to 0.34) | 496 (261 to 838) | 0.35 (0.18 to 0.58) | 153 (79 to 265) | 0.09 (0.04 to 0.15) |
| 2015 | 651 (346 to 1116) | 0.20 (0.11 to 0.34) | 497 (261 to 843) | 0.34 (0.18 to 0.59) | 154 (76 to 265) | 0.09 (0.04 to 0.15) |
| 2016 | 652 (347 to 1105) | 0.20 (0.10 to 0.34) | 499 (259 to 841) | 0.34 (0.17 to 0.58) | 154 (76 to 265) | 0.08 (0.04 to 0.15) |
| 2017 | 655 (347 to 1108) | 0.19 (0.10 to 0.33) | 502 (257 to 848) | 0.33 (0.17 to 0.57) | 152 (75 to 262) | 0.08 (0.04 to 0.15) |
| 2018 | 656 (346 to 1110) | 0.19 (0.10 to 0.32) | 505 (264 to 855) | 0.33 (0.17 to 0.56) | 151 (73 to 264) | 0.08 (0.04 to 0.14) |
| 2019 | 659 (344 to 1131) | 0.19 (0.10 to 0.32) | 508 (266 to 878) | 0.32 (0.17 to 0.55) | 150 (71 to 265) | 0.08 (0.04 to 0.14) |
| 2020 | 663 (344 to 1139) | 0.19 (0.10 to 0.32) | 511 (264 to 875) | 0.32 (0.17 to 0.54) | 152 (72 to 271) | 0.08 (0.04 to 0.14) |
| 2021 | 669 (337 to 1145) | 0.19 (0.10 to 0.32) | 519 (266 to 879) | 0.32 (0.17 to 0.54) | 150 (72 to 264) | 0.08 (0.04 to 0.14) |
| DALYs, disability-adjusted life years. UI, uncertainty interval. | | | | | | |

| Table S13. Arima projections of age-standardized rate of burden for vision impairment due to age-related macular degeneration prevalence, 2022–2040 | | | | | | |
| --- | --- | --- | --- | --- | --- | --- |
|  | | Sex | | |  | |
| Year | Both | | Male | Female | |  |
| 1990 | 22.3 | | 21.8 | 22.6 | | Actual |
| 1991 | 22.0 | | 21.6 | 22.2 | | Actual |
| 1992 | 21.7 | | 21.4 | 21.9 | | Actual |
| 1993 | 21.5 | | 21.2 | 21.7 | | Actual |
| 1994 | 21.3 | | 21.0 | 21.4 | | Actual |
| 1995 | 21.1 | | 20.9 | 21.3 | | Actual |
| 1996 | 21.0 | | 20.8 | 21.1 | | Actual |
| 1997 | 20.9 | | 20.7 | 21.0 | | Actual |
| 1998 | 20.8 | | 20.6 | 20.9 | | Actual |
| 1999 | 20.7 | | 20.6 | 20.8 | | Actual |
| 2000 | 20.7 | | 20.5 | 20.7 | | Actual |
| 2001 | 20.6 | | 20.3 | 20.7 | | Actual |
| 2002 | 20.4 | | 20.2 | 20.6 | | Actual |
| 2003 | 20.3 | | 20.0 | 20.5 | | Actual |
| 2004 | 20.2 | | 19.8 | 20.5 | | Actual |
| 2005 | 20.1 | | 19.7 | 20.4 | | Actual |
| 2006 | 20.0 | | 19.6 | 20.3 | | Actual |
| 2007 | 19.9 | | 19.6 | 20.1 | | Actual |
| 2008 | 19.8 | | 19.5 | 20.0 | | Actual |
| 2009 | 19.7 | | 19.5 | 19.9 | | Actual |
| 2010 | 19.7 | | 19.4 | 19.8 | | Actual |
| 2011 | 19.7 | | 19.4 | 19.8 | | Actual |
| 2012 | 19.8 | | 19.5 | 19.9 | | Actual |
| 2013 | 19.8 | | 19.5 | 20.1 | | Actual |
| 2014 | 19.9 | | 19.5 | 20.2 | | Actual |
| 2015 | 20.0 | | 19.5 | 20.3 | | Actual |
| 2016 | 19.9 | | 19.5 | 20.2 | | Actual |
| 2017 | 19.9 | | 19.5 | 20.1 | | Actual |
| 2018 | 19.8 | | 19.4 | 20.0 | | Actual |
| 2019 | 19.7 | | 19.4 | 20.0 | | Actual |
| 2020 | 19.7 | | 19.2 | 20.0 | | Actual |
| 2021 | 19.7 | | 19.4 | 19.9 | | Actual |
| 2022 | 19.8 | | 19.5 | 19.6 | | Forecast |
| 2023 | 19.93 | | 19.56 | 19.3 | | Forecast |
| 2024 | 20.06 | | 19.62 | 19.01 | | Forecast |
| 2025 | 20.19 | | 19.68 | 18.71 | | Forecast |
| 2026 | 20.3 | | 19.74 | 18.42 | | Forecast |
| 2027 | 20.38 | | 19.8 | 18.12 | | Forecast |
| 2028 | 20.43 | | 19.86 | 17.83 | | Forecast |
| 2029 | 20.47 | | 19.92 | 17.53 | | Forecast |
| 2030 | 20.5 | | 19.98 | 17.24 | | Forecast |
| 2031 | 20.54 | | 20.04 | 16.95 | | Forecast |
| 2032 | 20.6 | | 20.1 | 16.65 | | Forecast |
| 2033 | 20.67 | | 20.16 | 16.36 | | Forecast |
| 2034 | 20.75 | | 20.22 | 16.06 | | Forecast |
| 2035 | 20.84 | | 20.28 | 15.77 | | Forecast |
| 2036 | 20.92 | | 20.34 | 15.47 | | Forecast |
| 2037 | 21 | | 20.4 | 15.18 | | Forecast |
| 2038 | 21.07 | | 20.46 | 14.88 | | Forecast |
| 2039 | 21.14 | | 20.52 | 14.59 | | Forecast |
| 2040 | 21.2 | | 20.58 | 14.29 | | Forecast |
| DALYs, disability-adjusted life years. UI, uncertainty interval. | | | | | | |


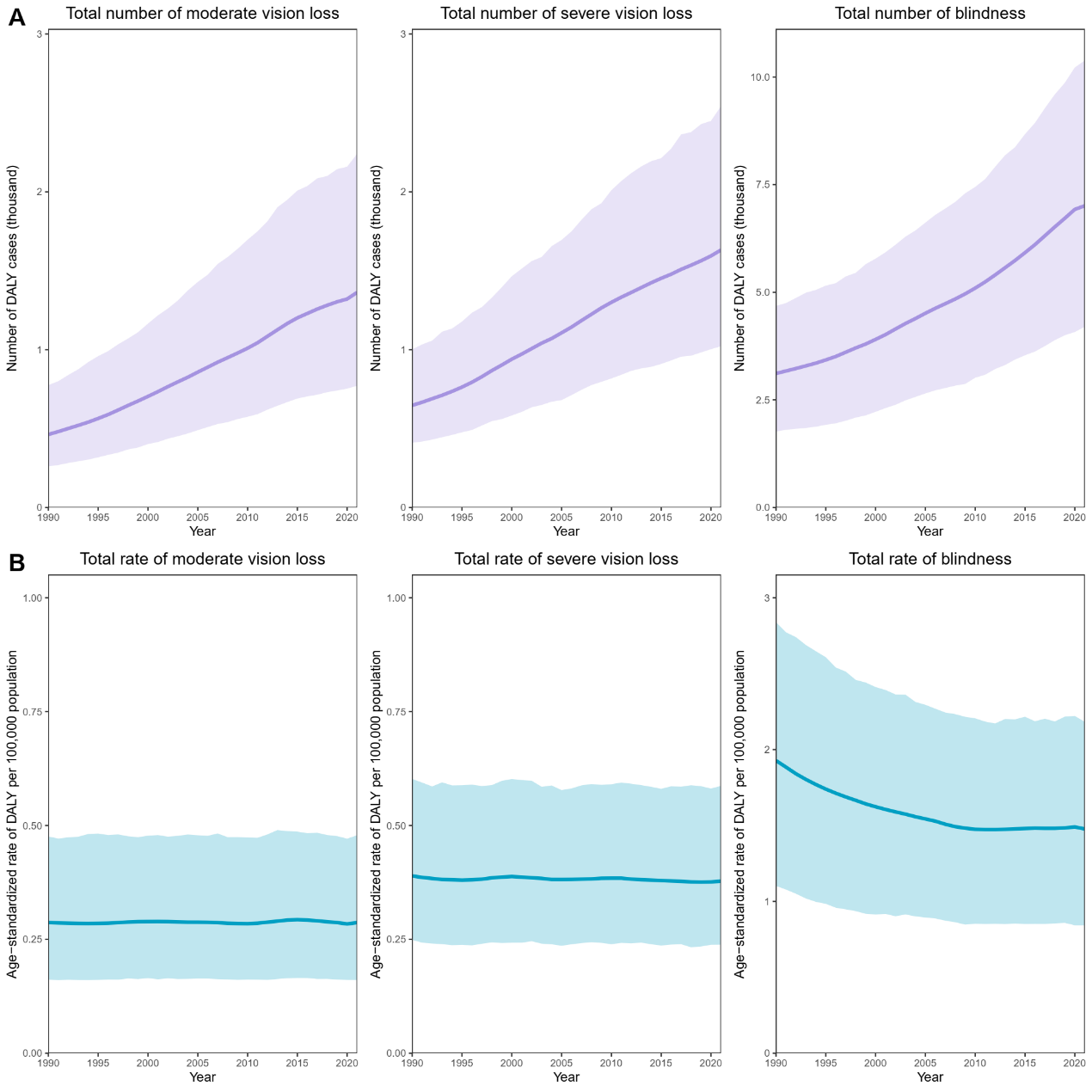


Figure S1. Vision impairment due to age-macular degeneration by constituent sequelae DALY for both sexes combined, 1990–2021. (A) Total number of moderate vision loss, severe vision loss, and blindness. (B) Rates (per 100,000 population) of age-standardized moderate vision loss, severe vision loss, and blindness. Shaded regions indicate 95% uncertainty intervals. DALY: disability-adjusted life-years.


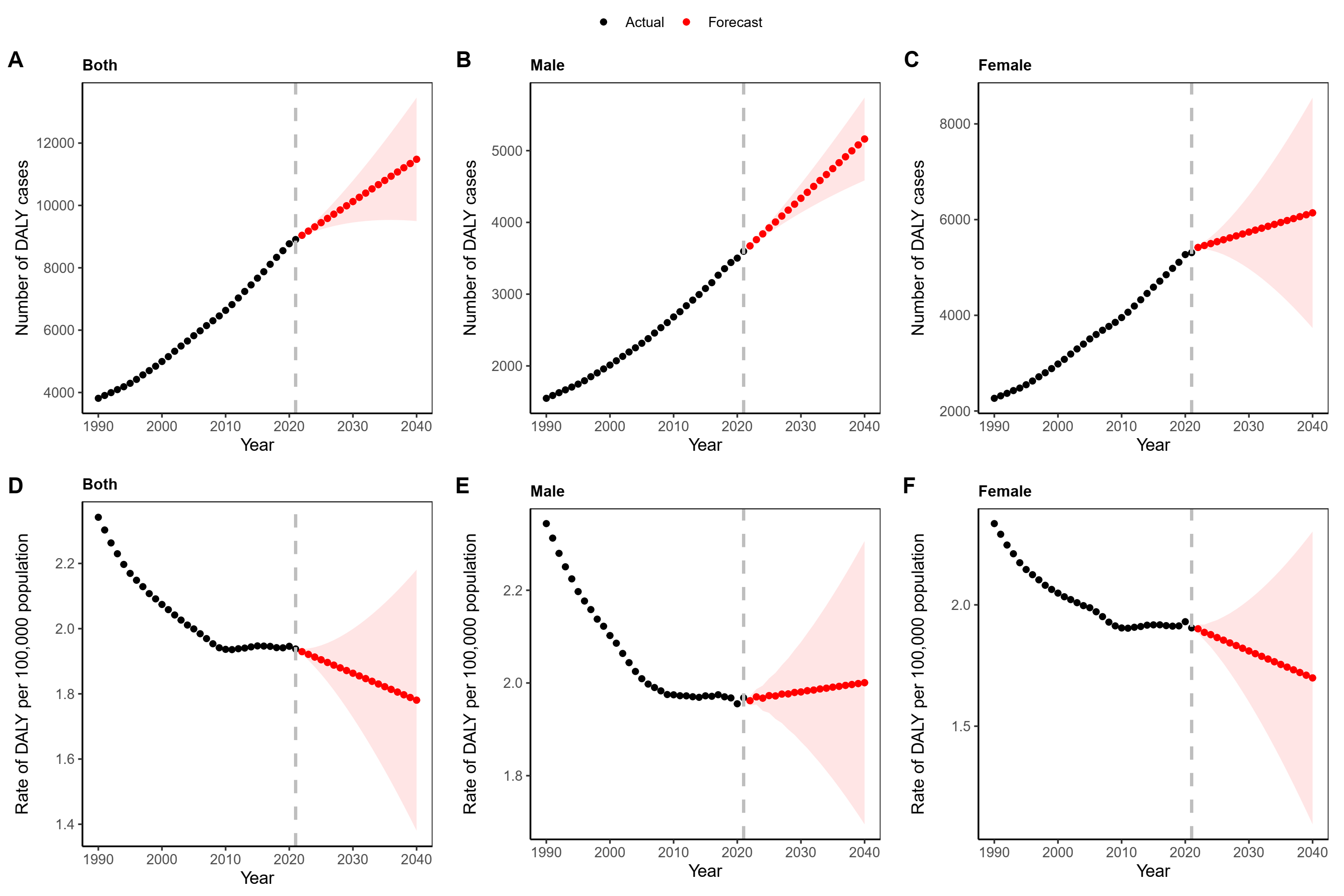


Figure S2. Projections of burden of vision impairment due to age-related macular degeneration DALY by sex, 2022–2040. Shaded regions indicate 95% confidence intervals. DALYs, disability-adjusted life years.

(A) Predictions of the cases of DALY from 2022 to 2040 (Both)

(B) Predictions of the cases of DALY from 2022 to 2040 (Male)

(C) Predictions of the cases of DALY from 2022 to 2040 (Female)

(D) Predictions of the age-standardized DALY rate from 2022 to 2040 (Both)

(E) Predictions of the age-standardized DALY rate from 2022 to 2040 (Male)

(F) Predictions of the age-standardized DALY rate from 2022 to 2040 (Female)
